# 2D Copper MOF Membranes with Precise Pores for Ionic Memory

**DOI:** 10.1002/advs.202516193

**Published:** 2026-03-24

**Authors:** Hiran Jyothilal, Raj Kumar Gogoi, Yuchen Liu, Vikas Sharma, Solleti Goutham, Michael Hutton, Rachel C. Kilbride, Daniel T.W. Toolan, Ashok Keerthi, Boya Radha

**Affiliations:** ^1^ Department of Physics and Astronomy The University of Manchester Manchester UK; ^2^ National Graphene Institute The University of Manchester Manchester UK; ^3^ Department of Chemistry The University of Manchester Manchester UK; ^4^ Department of Materials The University of Manchester Manchester UK; ^5^ Department of Physics The University of Warwick Coventry UK; ^6^ XMaS The UK X‐ray Materials Science Facility European Synchrotron Radiation Facility Grenoble France; ^7^ Photon Science Institute The University of Manchester Manchester UK

**Keywords:** ionic memristor, MOF, nanofluidics, osmotic energy, ultrathin 2D membranes

## Abstract

Biological ion channels play a critical role in the complex physiological processes of living organisms. Inspired by these natural systems, we demonstrate the fabrication of ultrathin 2D copper‐based metal‐organic framework (CuMOF) films and their application to mimic key functionalities of biological membranes. Using a liquid–air interface synthesis technique, we synthesized CuMOF films with tunable thicknesses ranging from 1.4 nm to 20 nm over centimeter‐scale areas. These membranes exhibit well‐defined nanoporous structures of pore diameter 1.5 nm that facilitate nanofluidic ion transport. The ionic conductance of the CuMOF films strongly depends on both film thickness and electrolyte concentration, demonstrating surface‐charge‐dominated transport and Na^+^ selective ion conduction at low ionic strengths. Moreover, this selective ion transport enables osmotic energy generation under a concentration gradient. Additionally, the CuMOF films exhibit ionic memristive behavior, including reversible synaptic potentiation and depression, closely resembling synaptic plasticity. This study presents a scalable, tunable platform for developing bioinspired ionic devices with potential applications in selective ion transport, nanofluidic energy harvesting, and neuromorphic computing.

## Graphical abstract

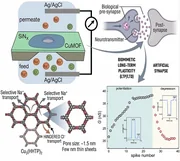

## 1 Introduction

Cell membranes are semipermeable protein membranes with well‐defined ion channels that facilitate the selective transport of ions across the membrane. They are essential for maintaining the cell's electrochemical gradient and play a critical role in various physiological processes such as selective ion transport, cellular communication, homeostasis, and energy generation [1, 2, 3]. Inspired by these natural channels, artificial ion channels are being developed, offering a diverse array of applications including selective ion‐transport, ion pumping, ionic‐memory, electrochemical energy generation, DNA/RNA sequencing, neural signaling and targeted drug delivery [4, 5, 6, 7, 8, 9, 10, 11, 12, 13, 14, 15]. A diverse range of ion channels, such as single nanopores, slit‐shaped 2D channels, and nanoporous membranes, has been fabricated to mimic the fascinating properties of their biological counterparts [16, 17, 18]. Although in most of these artificial channels, the channel diameter is similar to that of biological channels [19] (< 2 nm) has been successfully achieved, this often comes at the cost of increased channel length or thickness (∼5 to 10 nm for biological channels). Recent efforts achieved ultra‐thin channel lengths using 2D materials such as MoS_2_ and graphene, often requiring highly sophisticated fabrication techniques [4, 20, 21]. MOF membranes can be fabricated using straightforward solution‐based, interfacial, or vapor‐assisted methods. Specifically, continuous MOF membranes have been synthesized by solvothermal and interfacial strategies [22], ultrathin MOF films have been produced via scalable gel–vapor deposition [23] and COF membranes have been assembled using aqueous two‐phase or oil–water interfacial polymerization techniques [24, 25, 26]. Herein, we demonstrate the fabrication of MOF films with thicknesses ranging from 1.4 nm to 20 nm to mimic certain functionalities of biological ion channels, such as selective ion transport, osmotic energy generation and ionic memristor.

MOFs are highly crystalline materials characterized by their high porosity and the periodic arrangement of metal centers within organic frameworks. The pore size and shape of these frameworks can be precisely tuned through the choice of organic linkers, ligands, and solvents, enabling control over their dimensionalities [27, 28]. This architectural tunability makes MOFs highly attractive for electrochemical energy applications and various other fields [29, 30, 31]. For example, chemically cross‐linked nanometal‐organic frameworks (NMOFs) exhibit high selectivity in CO_2_ adsorption over N_2_ and CH_4_, making them suitable for CO_2_ separation and conversion [32]. MOF‐based composite hydrogels have also shown promise in biomedical applications such as peripheral nerve repair, where functional peptides can be incorporated into the framework [33]. Despite these advantages, transforming MOFs from their typical crystalline powder form into macroscopic 3D structures remains a significant challenge due to their limited solubility, poor processability, and instability in certain chemical environments [34]. Fabricating thin MOF films offers a promising solution, enabling the creation of membranes with uniformly distributed pores that act as confined ion channels, ideal for selective ion transport [35]. Such ultrathin MOF membranes have demonstrated potential in water ion separation [36], enhanced water permeation [37, 38] and even solid‐state neuromorphic applications when integrated into resistive random‐access memory devices [39]. Although MOFs are continuously being explored for various applications, their use in scalable technologies requires these films to be scalable in lateral dimensions, possess tunable thickness, and be cost‐effective to produce [40]. In this study, we demonstrate the fabrication of free‐standing MOF membranes with tunable thickness. Depending on the intended application, such as ion selectivity, osmotic energy generation or ionic memory, our method of preparing MOF films offers the flexibility to tailor thickness according to specific functional requirements.

## 2 Synthesis and Structural Characterization of Ultrathin 2D CuMOF Films

**FIGURE 1:**
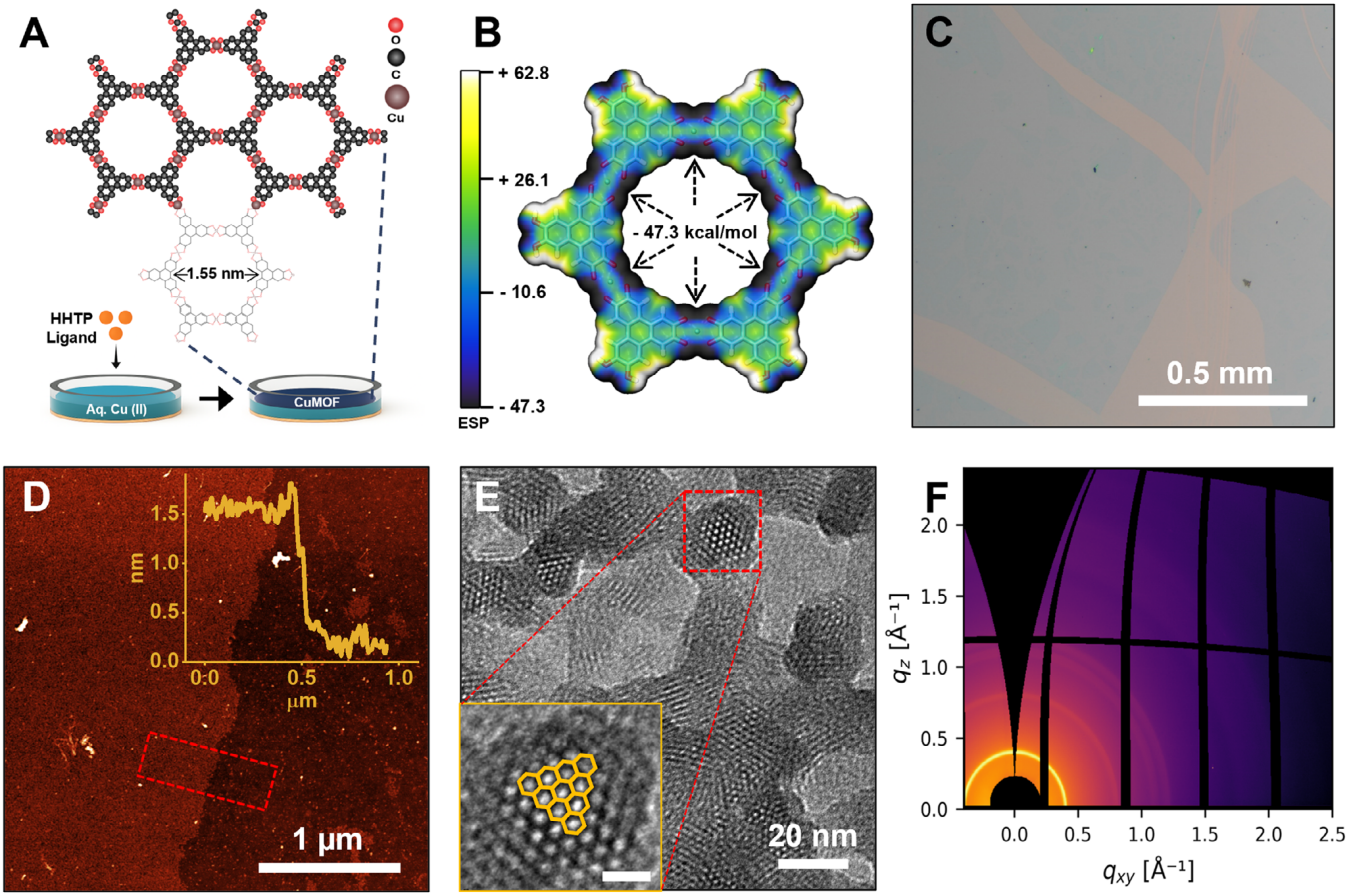
Facile synthesis method of ultrathin CuMOF membrane. (A) Schematics of CuMOF chemical structure with a pore size of approximately 1.55 nm and CuMOF synthesis process. CuMOF is then scooped onto the desired substrate. (B) Electrostatic potential (ESP) analysis of CuMOF with a minimum potential of −47.3 kcal/mol at the O─Cu─O site. (C) Large area optical image of 2.4 nm thick CuMOF film scooped onto a SiO_2_ substrate. (D) AFM image of CuMOF ultrathin membrane, ∼1.4 nm thick (inset: height profile from CuMOF edge. Profile enlarged for better visibility). (E) HRTEM image of 20 nm thick CuMOF membrane showing ring structure (Inset: magnified image, scalebar 5 nm). (F) 2D GIWAXS pattern of a CuMOF membrane.

Ultrathin CuMOF films (structure in Figure 1A) were synthesized via liquid‐air interface synthesis technique [41]. We use 2, 3, 6, 7, 10, 11‐hexahydroxy triphenylene (HHTP) as the ligand and Cu (II) has been used as the metal center. First, a 2 mm ligand solution was prepared by dissolving HHTP in a solvent mixture containing benzaldehyde, ethyl acetate and ethanol (9:9:2 volume ratio) in the required molarity. For the metal source, an aqueous 5 mm copper (II) acetate [Cu(CH_3_COO)_2_] solution was prepared. To perform the liquid‐air interface synthesis of the CuMOF films, the Cu(CH_3_COO)_2_ solution was taken in a petri dish and 10 µL of the ligand solution was added dropwise (schematically shown in Figure 1A). A pale blue CuMOF film is formed instantaneously on the liquid‐air interface (Figure S4) and was carefully scooped onto the desired substrate within 10 s of the ligand‐addition (Figure 1C). The film was then washed multiple times with acetone, ethanol, and deionized water to remove residual ligands, organic solvents, and excess copper acetate, followed by drying with a gentle N_2_ gas flush. The thinnest CuMOF film synthesized is ∼1.4 nm thick and the most reproducible thickness is 2.4 nm (AFM image in Figure 1D). High‐resolution transmission electron microscopy (HRTEM) images were acquired by scooping CuMOF films onto holey carbon TEM gold grids (Figure 1E). The HRTEM image confirms the formation of ∼1.55 nm diameter columnar porous structure of CuMOF, which can facilitate mass transport by using ion channels. For TEM‐based measurements, 20 nm thick CuMOF films were used as they sustain electron beam for a longer duration. The Grazing incidence wide‐angle X‐ray scattering pattern (GIWAXS) of a 165 nm thick CuMOF film shows (Figure 1F and Figure S8) strong Debye‐Scherrer rings at 0.40, 0.80, 1.09 and 1.19 Å^−1^, corresponding to d‐spacings of 1.55, 0.78, 0.57, and 0.53 nm, respectively. As such, the 165 nm CuMOF crystallites are randomly oriented with respect to the substrate surface. The d‐spacing of the first order scattering feature is commensurate with that of the predicted pore diameter of CuMOF 1.55 nm, so we ascribe the features at 0.40 and 0.80 Å^−1^ as (100) and (200) features.

**FIGURE 2:**
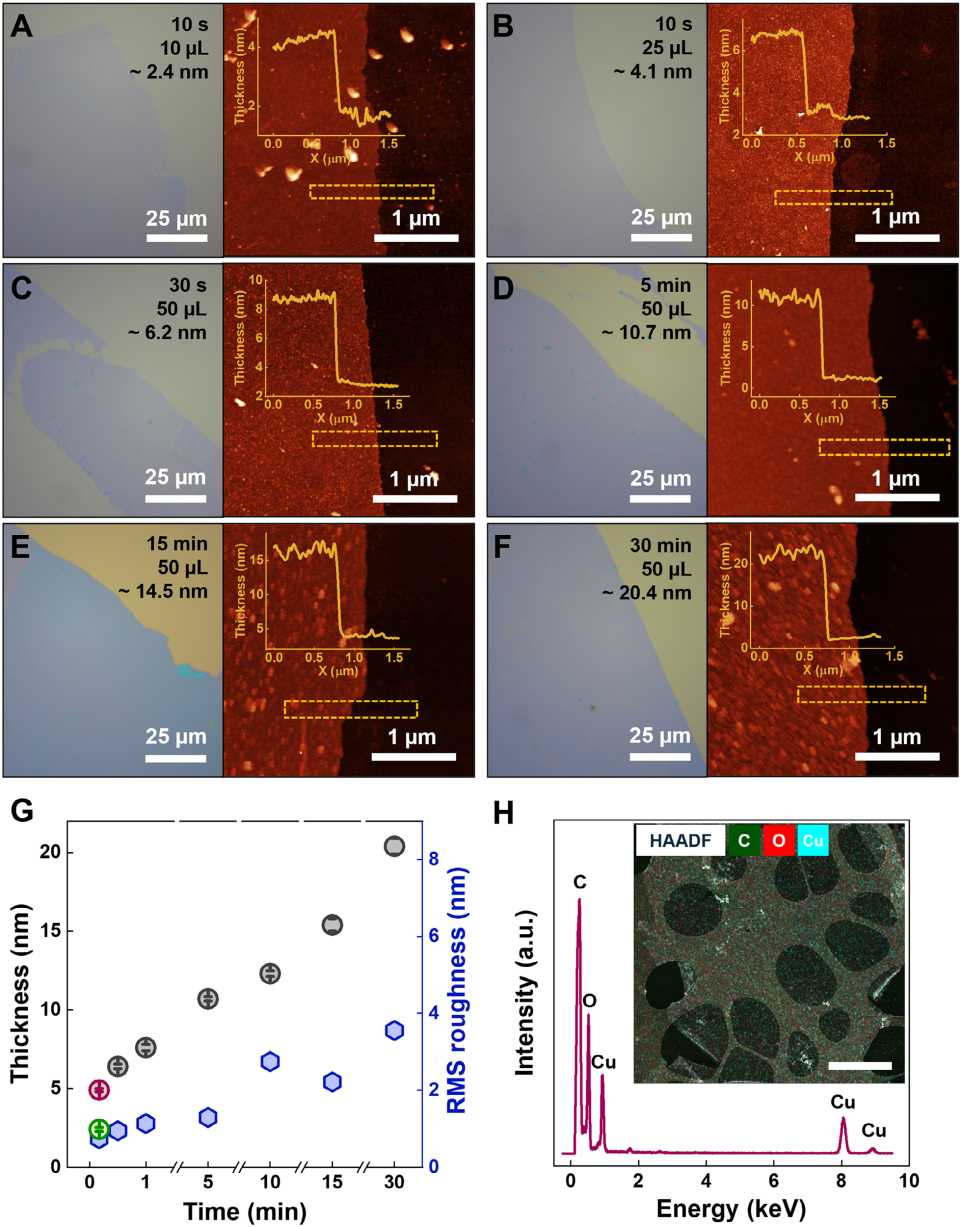
Tunable thickness of CuMOF films and elemental composition. Optical contrast and AFM thickness measurements of synthesized CuMOF films using various combinations of ligand volume and synthesis time. (A–F) Optical and AFM images of MOF synthesized using different parameters such as synthesis time and ligand volume (insets showing AFM profiles of synthesized film thicknesses. Profile enlarged for better visibility). (G) Variation of film thickness (with error bars) and surface roughness of CuMOF with respect to the scooping time onto Si/SiO_2_ substrates. Green and red data points represent CuMOF film synthesized using 10 and 25 µL, respectively. (H) EDS spectra of freestanding CuMOF membrane on holey carbon TEM grid depicting the elemental composition peaks of its composite elements such as carbon, oxygen and copper (Inset; HAADF‐STEM EDS mapping showing elemental distribution, scalebar: 2 µm).

The thickness of CuMOF (*T*_CuMOF_) primarily depends on the volume of ligand solution used and the time waited before scooping [42]. Increasing the ligand volume from 10 to 25 µL, the *T*_CuMOF_ increases from 2.4 nm (Figure 2A) to 4.1 nm (Figure 2B) even when the reaction time is fixed at 10 s. Moreover, thicker films can be obtained by increasing the volume of the ligand and the scooping time. Increasing the ligand volume to 50 µL and increasing the reaction time from 30 s to 30 min, the *T*_CuMOF_ increases from 6.2 to 20.4 nm. Figure 2C–F illustrate the optical microscopic images alongside the corresponding AFM images and thickness profiles of the synthesised CuMOF film. The optical images clearly show a progressive increase in contrast with increasing *T*_CuMOF_. AFM surface morphology analysis (Figure 2G) further confirms that surface roughness increases with thickness, however, the film thickness to surface roughness ratio also decreases with increasing thickness, indicating the growth of highly ordered nanocrystalline domains (Figure S5). Scanning electron microscopy (SEM) image (Figure S6) shows the uniform film of CuMOF. High‐angle annular dark‐field scanning transmission electron microscopy (HAADF‐STEM) imaging (Figure S7) of a CuMOF film transferred onto a holey carbon support membrane demonstrates the CuMOF covering a large area. Elemental composition and distribution of Cu, C, and O were confirmed via energy‐dispersive X‐ray spectroscopy (EDS) and elemental mapping (Figure 2H). Gold TEM grids with holey carbon support films were specifically chosen over conventional Cu grids to eliminate background Cu signals. X‐ray diffraction (XRD) measurements were performed using grazing incidence XRD (GIXRD) to further characterise the crystalline structure of the CuMOF films [41, 43, 44] and the results back the GIWAXS data (Figure S10). This time‐ and volume‐dependent growth process demonstrates excellent tunability and reproducibility in controlling the thickness of CuMOF film. X‐ray photoelectron spectroscopy (XPS) analysis was performed to confirm the chemical composition, oxidation states and coordination environment of the Cu‐MOF thin films (Figure S11). Survey spectra show the expected elemental signatures of Cu, C and O, consistent with the organic linker and the metal–node structure of the framework (Figure S11A). There were no signals associated with contamination or precursor residues, elucidating high chemical purity of the synthesized CuMOF ultrathin films. High‐resolution Cu 2p spectra display two principal peaks at Cu 2p_3/2_ and Cu 2p_1/2_, accompanied by characteristic satellite features (Figure S11B) [45, 46]. The presence of shake‐up satellites indicates a Cu^2^^+^‐dominated oxidation state. The relative satellite intensity and the fitted binding energies confirm that the synthesis process preserves the oxidation state required for redox‐active electronic behavior in neuromorphic operation. The O 1s region reveals contributions from Cu─O coordination between copper and oxygen in the HHTP ligand (Figure S11C). The C 1s spectrum contains contributions from aromatic carbon, such as C─C/C─H, C─OH and C═O groups, which are characteristic of the HHTP ligand (Figure S11D) [47].

## 3 Ionic Transport Properties of Ultrathin 2D CuMOF Membranes

**FIGURE 3:**
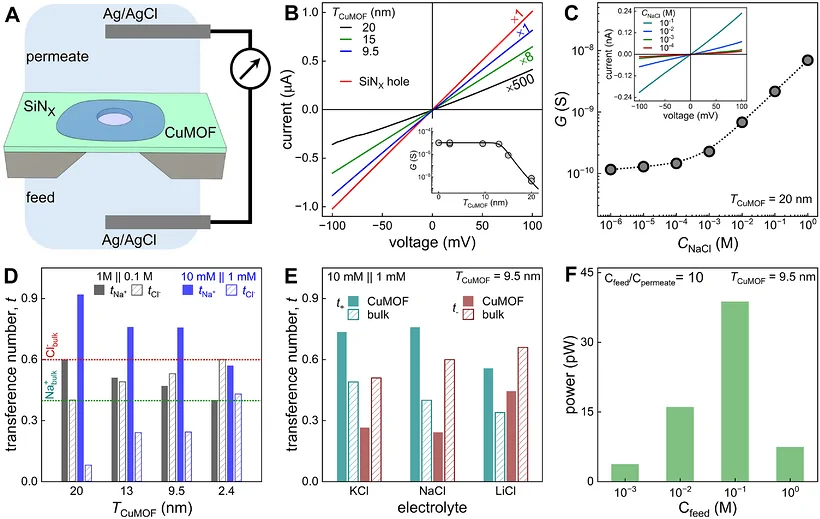
Selective‐ion transport through CuMOF nanoporous films. (A) Schematic of the experimental setup used to measure ion transport. (B) The current vs voltage (*I‐V*) curves through a SiN_X_ micro‐hole (hole diameter, 2 µm) and MOF nanoporous films (film thickness, 2.4, 9.5, 13, 15, and 20 nm) covering a SiN_X_‐hole (termed CuMOF devices). The inset in (B) compares the conductance (*G*) of bare micro‐hole and CuMOF devices. In (B) 1 M NaCl is used as an electrolyte. (C) *G* of a CuMOF device (*T*_CuMOF_ of 20 nm) as a function of NaCl concentration (*C*_NaCl_). The inset shows *I‐V* curves at varying *C*_NaCl_. (D) Transference numbers (*t*) of CuMOF devices with different film thicknesses measured under NaCl concentration gradients of 1 M || 0.1 M and 10 mm || 1 mm (*ΔC* = 10). The dotted lines refer to bulk *t* of Na^+^ and Cl^−^ ions as green and dark red colors respectively. (E) Comparison of t for a 9.5 nm‐thick CuMOF device under 10 mm || 1 mm concentration gradients of NaCl, KCl, and LiCl electrolytes. (F) Osmotic power output from a CuMOF device (*T*_CuMOF_ of = 9.5 nm) at different NaCl concentration gradients (*ΔC* = 10). In (D–F), the *C*_NaCl_ in the reservoir containing the counter electrode (CE) is maintained at a higher concentration than that in the reservoir with the working electrode (WE).

To mimic the functionalities of the biological membranes, the ion transport properties of the nanoporous CuMOF membranes were studied by placing the as synthesized CuMOF films on top of a pre‐made SiN_X_ micro‐hole (termed as CuMOF device, schematically shown in Figure 3A). The CuMOF device was sandwiched between two electrolyte reservoirs, each containing custom‐made Ag/AgCl electrodes immersed in the reservoirs (Figure 3A) and connected to a Keithley 2636B source meter to record the electrochemical characteristics. The voltage‐dependent ionic current (*I‐V*) of different CuMOF devices (film thickness, *T*_CuMOF_ of 9.5, 15, and 20 nm), recorded with 1 M NaCl as the electrolyte in both reservoirs, produced *I‐V* curves (Figure 3B) with a finite conductance (*G* = *I/V*). The *G* of these CuMOF devices is lower than that of the SiN_X_ micro‐hole (diameter, 2 µm), confirming the coverage of hole with the CuMOF films. The decrease in *G* is dependent on the *T*_CuMOF_, shown in the inset of Figure 3B. As the *T*_CuMOF_ increases, the channel lengths increase and the quality of the films improves, leading to a reduction in defects (confirmed by the surface roughness measurements, Figure 2G). To study the nanofluidic ion transport of the CuMOF nanochannels, we estimate the concentration (*C*) dependent *G* of the CuMOF devices from the *I‐V* curves recorded across the CuMOF device using a range of *C*_NaCl_ (10^−6^ M – 1 M). The *G* *vs* *C* curve in Figure 3C reveals two distinct regimes for the device of *T*_CuMOF_ 20 nm, *G* remains independent of *C* for *C*_NaCl_ < 10^−3^ M, while *G* α *C* for *C*_NaCl_ ≥ 10^−3^ M. These types of *G* *vs* *C* graphs are characteristic of confined fluidic channels with surface charges, where the *G* in the low‐concentration regime is primarily governed by the surface‐charge‐density of the channels rather than the concentration in the reservoirs [20, 48]. The presence of the plateau in the *G* vs *C* graph indicates the preferential transport of particular charged ions over the others.

The preferential flow of ions was investigated by measuring the *I‐V* curves while maintaining concentration gradients (Δ*C*) across the CuMOF device. With different *C*_NaCl_ (2 mL in each reservoir) and Δ*C* = 10, the *I‐V* curves exhibit different intercepts on the axis (Figure S14A); the intercept in the voltage‐axis gives the zero‐current potential (i.e., total potential, *E*_T_) from which the selectivity of the ions has been estimated. Since the experiments were performed with custom‐made Ag/AgCl electrodes, the electrode contributions (i.e., redox potential, *E*_R_) should be removed to obtain the true potential (*E*_M_) [49], given by:

$$E_{M}=E_{T}-E_{R}$$

Whereas the *E*_R_ [49] is estimated as:

$$E_{R}=\frac{RT}{nF}ln\left(\frac{\gamma_{H}C_{H}}{\gamma_{L}C_{L}}\right)$$

where *F* is the Faraday constant; *R* is the universal gas constant; *T* is the temperature; *n* is the number of electrons involved in the reaction; *γ*_H/L_ and *C*_H/L_ are the mean activity coefficient of electrolytes and corresponding concentration in the feed/permeate reservoirs, respectively. With the increase in *C*_NaCl_, the *E*_M_ decreases even when maintaining a fixed Δ*C* = 10, as compared in Table S1. The ions’ selectivity in terms of transference number, *t* is calculated from the *E*_M_ as:

$$E_{M}=\left(2t_{Na^{+}}-1\right)\;\frac{RT}{nF}ln\left(\frac{\gamma_{H}C_{H}}{\gamma_{L}C_{L}}\right)$$

At all *C*_NaCl,_ the CuMOF film with a thickness of 20 nm exhibits $t_{Na^{+}}>\;t_{Cl^{-}}$, indicating the Na^+^ selectivity in the channels, Figure S14B. However, the $t_{Na^{+}}$ decreases with the increase in the *C*_NaCl_. This occurs because, at low ionic strength, ion selectivity is stronger due to surface‐charge‐dominated transport. As the concentration increases, bulk ion transport dominates, reducing the selectivity effect. Moreover, the selectivity of these CuMOF devices is also dependent on the film thickness: decreasing the *T*_CuMOF_ decreases the $t_{Na^{+}}/t_{Cl^{-}}$. We studied the *T*_CuMOF_ dependent selectivity by maintaining a Δ*C* = 10 at different *C*. Under a 1 M || 0.1 M gradient of NaCl, reducing the *T*_CuMOF_ from 20 to 2.4 nm causes the *E*_M_ to decrease from 11 to −12.5 mV, approaching the bulk *E*_M_ of a non‐selective membrane (Figure S17). This results in a decrease in the $t_{Na^{+}}$ from 0.6 to 0.4 (Figure 3D, black solid bars). However, at a lower concentration, 10 mm || 1 mm, the CuMOF devices remain Na^+^ selective, but the reduction of the *T*_CuMOF_ from 20 to 2.4 nm led to a decrease of the $t_{Na^{+}}$ from 0.92 to 0.57 (Figure 3D, blue solid bars). This decrease in the ion selectivity, i.e. *E*_M_ can be attributed to the depletion of the charge separation with a decrease in channel length *l*, resulting from concentration polarization [50, 51].

In addition, we investigate the cation selectivity of the CuMOF membranes. For this, we measured the *I‐V*s through a CuMOF device (*T*_CuMOF_ of 9.5 nm) under 10 mm || 1 mm gradients of KCl, NaCl and LiCl electrolytes, Figure S18A. The *E*_M_ was highest for NaCl at 29.6 mV, followed by KCl at 27.1 mV and LiCl at 6.5 mV (Figure S18B). The resulting cation transference number indicates the CuMOF is more selective toward Na^+^; the cation and anion transference numbers are compared in Figure 3D. Osmotic energy is generated by the CuMOF nanochannels when a Δ*C* is maintained across films. Ions begin to diffuse from the high‐concentration side to the low‐concentration side. Due to the negatively charged surface of the CuMOF, cations are preferentially transported over anions, leading to a net charge separation, which results in the development of a diffusion potential, or the *E*_M_. The resulting power, i.e., the osmotic power, can be estimated as: *P*_osm_  = (*E*_M_^2^×*G*)/4. The *E*_M_ and *G* of a CuMOF device (*T*_CuMOF_ of 9.5 nm) under a Δ*C* = 10 of different NaCl concentrations are compared in Figure S19A. As the concentration increases, the *E*_M_ decreases while G increases. The *P*_osm_ increases from 3.7 to 38.8 pW (Figure 3F) as the concentration increases from 1 to 100 mm, but further increases in concentration to 1 M led to a decline in power output, decreasing to 7.4 pW, highlighting the trade‐off between *E*_M_ and *G* at high *C*. The *P*_osm_ generated is significant and comparable to the osmotic energy produced by biological ion channels in nature [52]. This device generates a maximum power density of 12.34 Wm^−2^ under ten‐fold gradient of 100 || 10 mm NaCl Figure S19B.

**FIGURE 4:**
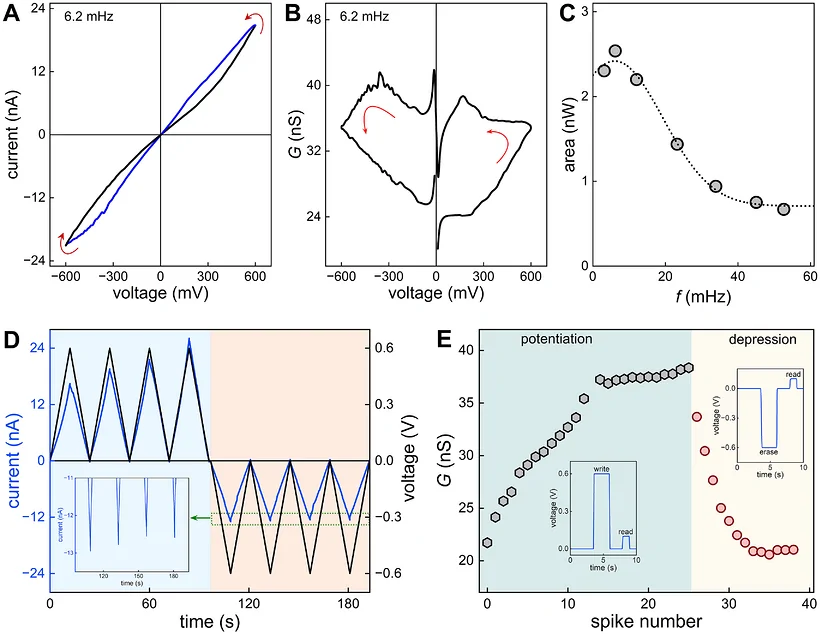
CuMOF‐based ionic memristor. (A) *I‐V* loops and (B) *G* vs *V* of a CuMOF device at a frequency of 6.2 mHz. (C) Frequency‐dependent area under the *I‐V* curves of a CuMOF device. (D) Ionic current response under voltage sweeps: positive voltage sweeps lead to a more dominant increase in ionic current, whereas negative voltage results in a slight decrease of ionic current in the subsequent pulses. (E) Potentiation followed by depression: a series of 25 write pulses (+0.6 V for 2 s, left inset) were applied, followed by 13 erase pulses (−0.6 V for 2 s, right inset), restoring the system to its initial state. The electrolyte used in this figure is 0.5 M NaCl and the CuMOF device is of thickness 20 nm.

The ability to retain and transmit information based on previous interactions is a fascinating property of biological membranes. These functional adaptations to past stimuli are made possible by the inherent ion channels in biological membranes, which act as memristors. To mimic this memristive property, different *I‐V* loops across the CuMOF devices were recorded at *C*_NaCl_ = 0.5 M. At a lower voltage range (± 0.2 V), the ionic currents during forward and backward sweeps were nearly overlapping. However, increasing the voltage range while maintaining a fixed scan rate (50 mV·s^−^^1^) resulted in a larger area under the curves, indicating the presence of two resistance states even for the same voltage (Figure S20). This observation confirms the presence of memristive behavior in CuMOF devices. The hysteresis observed in the *I‐V* curves arises from the history‐dependent distribution of ions under an applied bias, where the current at a given voltage is influenced by both the instantaneous electric field and the residual ion concentration from previous scans. Differences in the rates of ion transport during forward and backward voltage sweeps are governed by the adsorption and desorption of charge carriers on the channel walls [11, 53]. In confined nanochannels, mobile ions migrate and accumulate, generating non‐uniform concentration gradients that relax slowly upon voltage reversal, resulting in pinched hysteresis loops in both forward and reverse sweeps. This pinch hysteresis loop, characteristic of nanofluidic memristors, is a function of the scan rate [11, 53, 54, 55, 56]. In the CuMOF devices, the channels are selective for Na^+^ over Cl^−^, making Na^+^ the dominant mobile species. The memristive *I‐V* curves exhibit self‐crossing behavior (Figure 4A,B), and the frequency of the applied voltage strongly influences the hysteresis: higher frequencies produce smaller loops (Figure S21), whereas decreasing the frequency initially increases the loop area to a maximum (Figure 4C), beyond which further reduction reduces the area.

The CuMOF memristor was used to perform basic operations observed in biological synapses. First, a voltage was swept between zero and a fixed value while maintaining the same polarity to see the effect on the ion transport. Positive polarity sweeps led to an abrupt increase in ionic current with each cycle, whereas negative polarity resulted in a slight decrease (Figure 4D). We investigate the retention of a device by subjecting it to write pulses of 0.6 V of different width; 10, 30, 60, and 120 s. Increasing the pulse width enhances the potentiation (i.e., the change in *G*, insets in Figure S24) as well as the retention time. The pulse‐dependent potentiation and memory retention characteristics probe the concept of progression from transient synaptic enhancement to sustained synaptic strengthening. Synaptic potentiation and depression were demonstrated by applying sequential voltage pulses (Figure 4E). During potentiation, 25 write pulses (+0.6 V for 2 s), each followed by a rest pulse (0 V for 2 s) and a read pulse (+0.1 V for 1 s), led to a gradual increase in *G*, mimicking the potentiation in biological neurons. This suggests that the ions progressively accumulate or rearrange within the CuMOF channels, leading to a more conductive state under repeated stimulation. Following the potentiation phase, 13 erase pulses (−0.6 V for 2 s each), each followed by a rest pulse (0 V for 2 s) and a read pulse (+0.1 V for 1 s) were applied. The *G* at the read pulses gradually decreased, demonstrating synaptic weakening or depression, mimicking how biological synapses reduce their strength when exposed to inhibitory stimuli. This behavior demonstrates a controlled and reversible tuning of *G*, essential for information processing in neuromorphic computing.

To investigate the sodium ion adsorption behavior of CuMOF membrane, density functional theory (DFT) was employed. Initially, a fragment of CuMOF (Figure S25) was geometrically optimized (using the B3LYP/LANL2DZ method, applying B3LYP for the main group elements with 6–31G(d) basis set and LANL2DZ for copper). Next, a complete one‐ring unit was constructed based on the optimized geometries (Figure S25). The analysis revealed that the oxygen atoms carried the highest partial negative charge (−0.57) (Figure S26), and the electrostatic potential minima (−47.3 kcal/mol) were located at the O─Cu─O sites (Figure 1B), where lone pairs on oxygen atoms from adjacent ligands. Based on these findings, we concluded that sodium ions are likely to adsorb near the oxygen atoms, forming a square‐type arrangement with two oxygen atoms and one sodium ion around Cu^2+^ metal center. To further explore this interaction, the Na^+^CuMOF one‐ring structure was modeled by incorporating a Na^+^ ion into the CuMOF fragment, with the O‐Na^+^ bond distance calculated to be 2.09 Å. The charge on the adsorbed sodium ion was determined to be +0.75, lower than the charge of a free sodium ion (+1) due to the stabilization of nearby O─Cu─O moieties (Figure S26). This could be seen from the more positive charge from copper (from +0.66 to +0.84), and a more negative charge from the oxygen (−0.57 to −0.72). The adsorption/desorption Gibbs free energy was determined to be −166.5 kJ/mol per site, indicating a strong and favorable interaction driven by electrostatic attraction between the sodium ion and the CuMOF framework.

## 4 Conclusion

In summary, we have developed centimeter‐scale, tunable‐thickness CuMOF films that replicate key ion‐transport functions of biological membranes. The synthesis via liquid–air interface allows precise control over film thickness, critical for tuning ionic selectivity, conductance, and membrane behavior. Our findings reveal that these 2D CuMOF membranes can selectively transport cations, generate osmotic energy under salinity gradients, and mimic ionic memristive functions, including short‐term memory and synaptic plasticity. The thickness‐dependent selectivity and frequency‐sensitive memristive response highlight the versatility of these MOF‐based systems. This work opens new pathways for integrating ultrathin MOF films into functional ionic circuits for use in artificial synapses, energy harvesting, and advanced nanofluidic systems, thereby bridging the gap between synthetic and biological ion channel functionalities.

## Author Contributions

B.R. directed the project. B.R. and A.K. designed the project. H.J. synthesized CuMOF films, characterized them and fabricated CuMOF membrane devices and provided all the corresponding data. R.K.G. performed the electrochemical experiments with S.G. R.K.G. analyzed and provided all the electrochemical data. Y.L. performed and provided data for DFT studies and ESP analysis. V.S. synthesized and characterized the ligand, and provided the NMR data. D.T., R.C.K., and M.H. performed GIWAXS measurements and provided all the GIWAXS data. H.J., R.K.G., and B.R. wrote the manuscript. All authors contributed to the discussion.

## Conflicts of Interest

The authors declare no conflicts of interest.

## Supporting information




**Supporting File**: advs74921‐sup‐0001‐SuppMat.pdf.

## Data Availability

The data that support the findings of this study are available from https://doi.org/10.6084/m9.figshare.31704871.
