# Supplementary material for: 2D Copper MOF Membranes with Precise Pores for Ionic Memory
Source: Adv Sci (Weinh). 2026 Mar 24;13(31):e16193. doi: 10.1002/advs.202516193 (PMC13587955; doi:10.1002/advs.202516193)
Supplement: Supplementary file 1 [file ADVS-13-e16193-s001.pdf]

Supporting Information

2D Copper MOF membranes with Precise Pores for Ionic Memory

Hiran Jyothilal[1,2], Raj Kumar Gogoi[1,2], Yuchen Liu[3], Vikas Sharma[3], Solleti Goutham[1,2], Michael Hutton[4], Rachel C. Kilbride[5,6], Daniel T. W. Toolan[4], Ashok Keerthi[2,3,7]*, Boya Radha[1,2,7]*

[1]*Department of Physics and Astronomy, The University of Manchester, Manchester M13 9PL, UK*
[2]*National Graphene Institute, The University of Manchester, Manchester M13 9PL, UK*
[3]*Department of Chemistry, The University of Manchester, Manchester M13 9PL, UK*
[4]*Department of Materials. The University of Manchester, Manchester M13 9PL, UK*
[5]*Department of Physics, The University of Warwick, Coventry CV4 7AL, UK*
[6]*XMaS, The UK X-ray Materials Science Facility, European Synchrotron Radiation Facility, Grenoble, France*
[7]*Photon Science Institute, The University of Manchester, Manchester M13 9PL, UK*

Correspondence to ashok.keerthi@manchester.ac.uk; radha.boya@manchester.ac.uk

1. Synthesis and characterization of Hexahydroxy triphenylene (HHTP) ligand

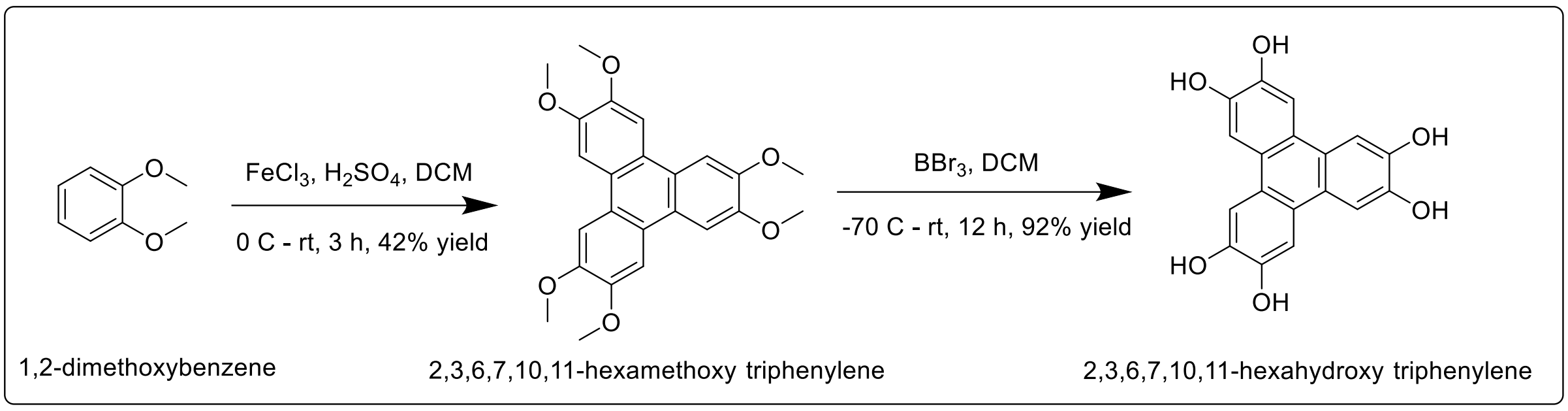

Scheme S1. Synthesis scheme for 2,3,6,7,10,11-hexahydroxy triphenylene.[1]

1.1. Synthesis of 2,3,6,7,10,11-hexamethoxy triphenylene

1,2-dimethoxybenzene (0.54 g, 3.9 mmol) was dissolved in dichloromethane (5 mL). In a separate flask, iron (III) chloride (1.9 g, 11.72 mmol) was suspended in dichloromethane (8 mL), and concentrated sulfuric acid (0.05 mL) was added dropwise at 0 °C. The solution containing the dissolved 1,2-dimethoxybenzene was incrementally added to the iron (III) chloride solution at 0 °C for a duration of 15 minutes. The resultant solution was permitted to gradually descend to room temperature over a duration of 3 hours while being stirred. Methanol was gradually introduced to quench the reaction (10 mL). The initial portion (3-4 mL) of methanol was introduced incrementally (dropwise) until the reaction transitioned from a viscous, tarry black slurry to a homogeneous opaque yellow suspension. After the whole addition of methanol, the reaction was agitated for 30 minutes and subsequently filtered. The white solid was meticulously washed with methanol and subsequently dried under reduced pressure, yielding pure hexamethoxy triphenylene (670 mg, 1.64 mmole, 42%).

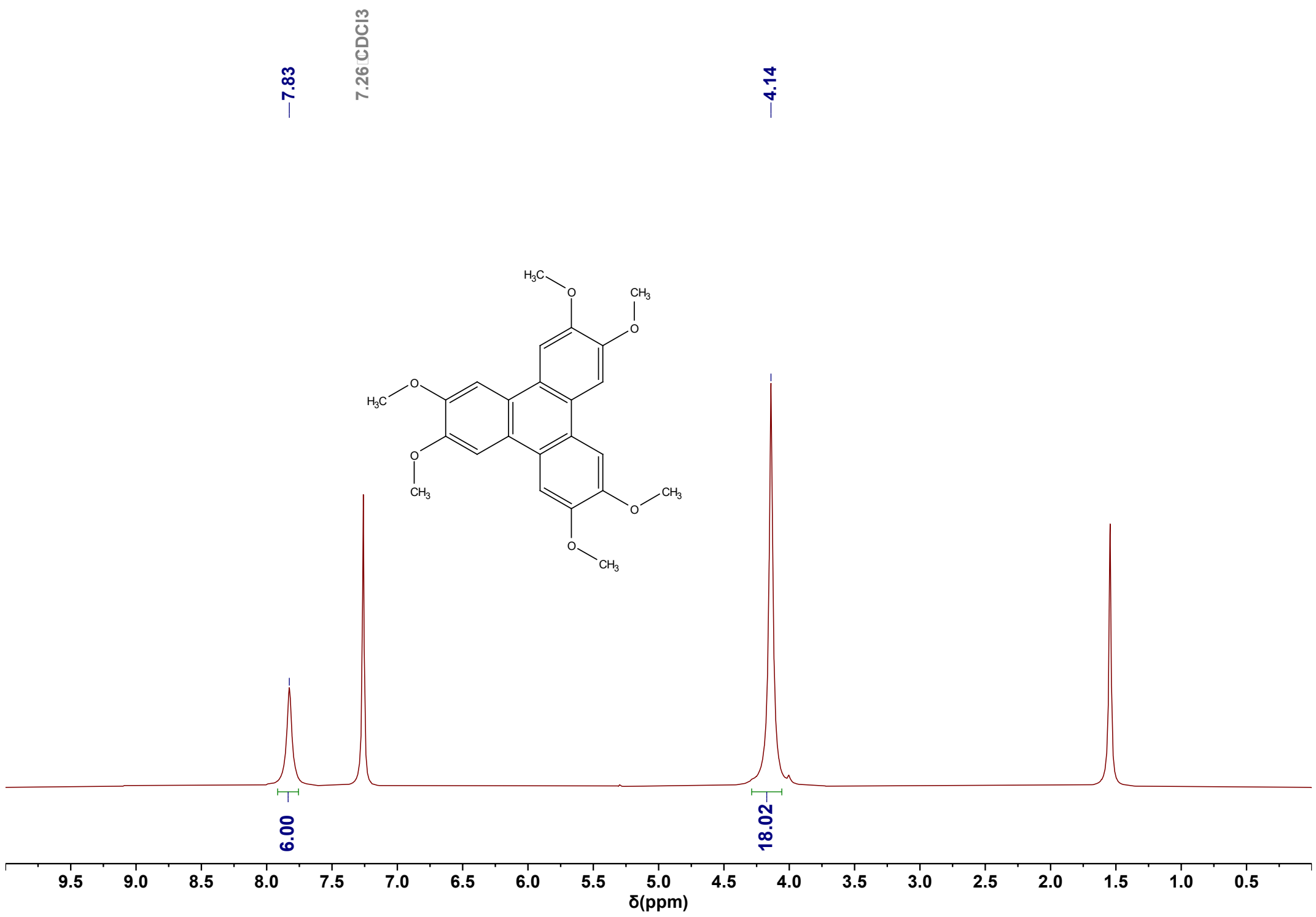

Fig. S1: ^{1}H NMR spectrum of 2,3,6,7,10,11-hexamethoxy triphenylene. ^{1}H NMR (500 MHz, $CDCl_3$): δ 7.83 (s, 6H), 4.14 (s, 18H).

1.2. Synthesis of 2,3,6,7,10,11-hexahydroxy triphenylene

Hexamethoxy-triphenylene (500 mg, 1.224 mmol) was dissolved in dichloromethane (40 mL) and the obtained solution was cooled to -70 °C and maintained under a nitrogen atmosphere. A solution of BBr_3 (1 M, DCM, 3 mL) was then added dropwise to the reaction mixture. After complete addition, the reaction temperature was gradually allowed to reach room temperature and stirring continued overnight. The reaction mixture was then slowly poured into crushed ice and the obtained mixture was stirred vigorously until the ice melted. It was then extracted with DCM and the combined organic extracts were washed with brine solution, dried over magnesium sulfate and evaporated to dryness to afford the pure 2,3,6,7,10,11-hexahydroxy triphenylene (368 mg, 1.134 mmol, 92%).

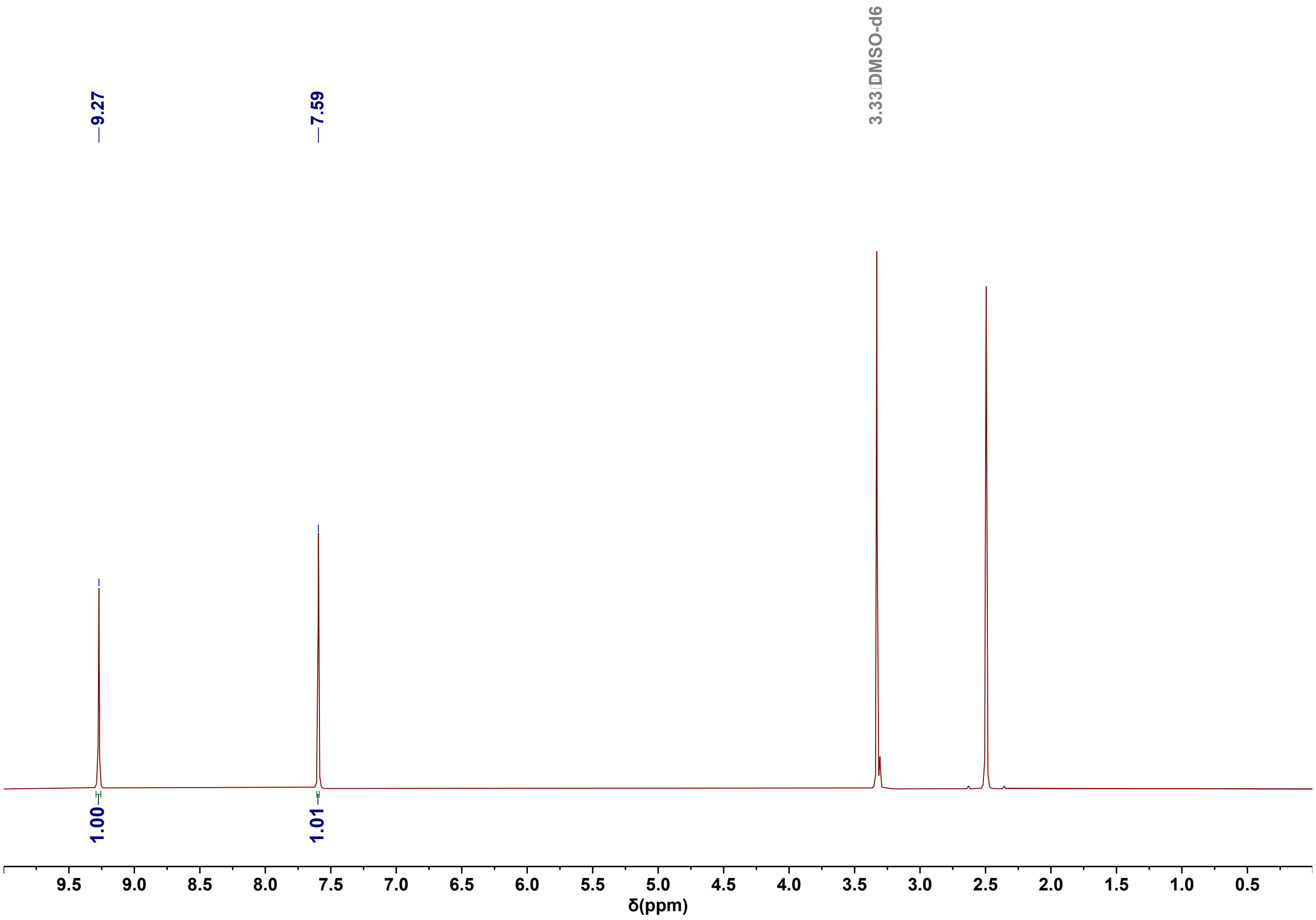

Fig. S2. ^{1}H NMR spectrum of 2,3,6,7,10,11-hexahydroxy triphenylene. ^{1}H NMR (500 MHz, DMSO) δ 9.27 (s, 6H), 7.59 (s, 6H).

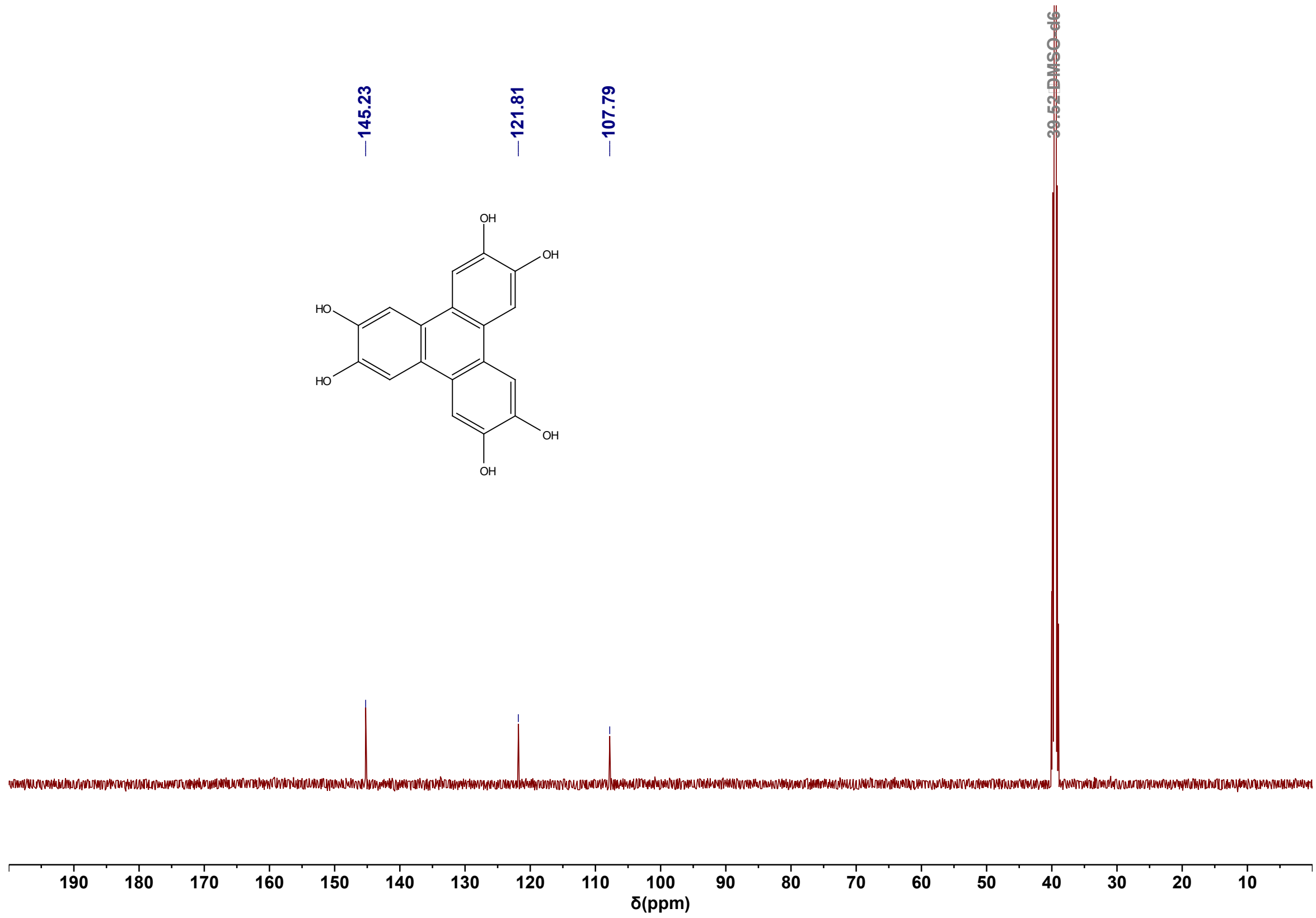

Fig. S3. ^{13}C NMR spectrum of 2,3,6,7,10,11-hexahydroxy triphenylene. ^{13}C NMR (126 MHz, DMSO) δ 107.79, 121.81, 145.23.

2. Fabrication of CuMOF membranes.

An aqueous solution of copper acetate 5 mM was made using commercially bought copper (II) acetate (Sigma Aldrich) and Millipore water. Ligand, 2,3,6,7,10,11-hexahydroxy triphenylene (HHTP), was synthesized in the lab (Scheme S1, Fig. S1-S3). Ligand solutions of various concentrations, such as 0.5 mM to 2 mM, were made by dissolving HHTP ligand in an organic solvent mixture of benzaldehyde, ethyl acetate and ethanol in a 9:9:2 ratio. Aqueous copper acetate solution was taken in a Petri dish in excess (20 - 50 mL) as a metal reservoir and ligand solution was injected onto the surface in a very low volume (10 – 50 μL) at ≈15 ± 2 °C under ambient conditions. The CuMOF membranes was instantaneously formed on the liquid-air interface. By varying the ligand concentration, volume and time of scooping to a substrate from the liquid-air interface, the thickness of the resultant CuMOF can be controlled. The interfacial membranes were gently scooped onto pre-cleaned substrates by fishing the substrate from underneath the membrane, to minimize mechanical stress and avoid contamination. The scooped CuMOF membrane was subsequently washed using acetone, ethanol and DI water, successively and dried under N_2 gas flow. Post-transfer washing and nitrogen drying steps were also explicitly included to ensure the removal of residual ligand and solvent residues.

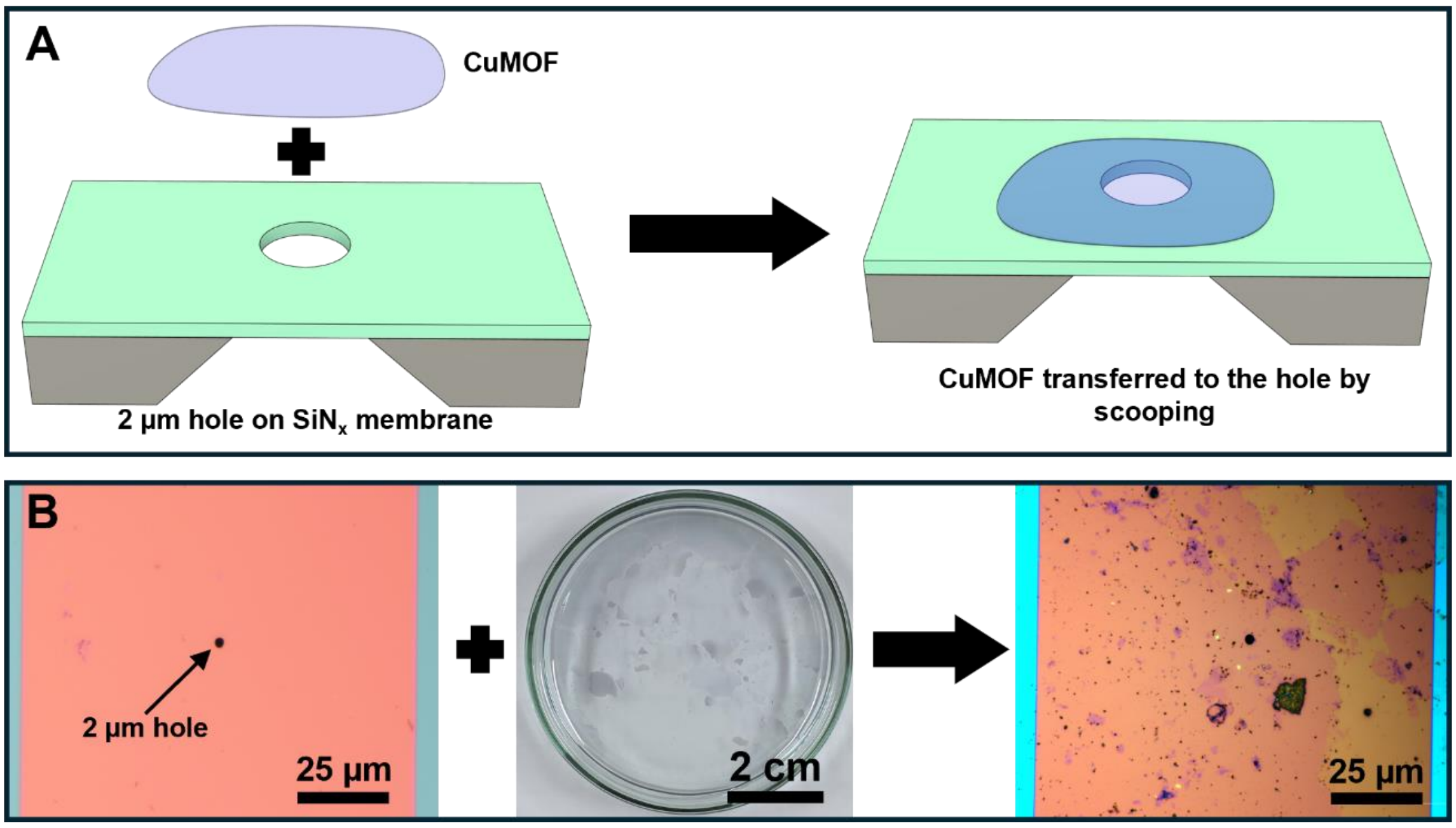

Fig. S4: Fabrication procedure of CuMOF membrane device. (A) Schematic representation of the fabrication procedure of the CuMOF device. (**B**) Optical images of the CuMOF device before and after scooping CuMOF membranes from the petridish.

3. Characterization of 2D CuMOF

CuMOF scooped onto the SiO_2 substrates were used for optical microscopy, SEM, XRD and AFM imaging purposes. Optical images were recorded using Nikon Eclipse compound optical Microscope. SEM images of CuMOF were taken on Zeiss Ultra FESEM instrument operated at 3 KV. XRD characterisation measurements were done on Pan Analytic XRD system. AFM imaging for thickness and surface roughness measurements was performed on Bruker FastScan AFM system. CuMOF scooped onto holey carbon gold TEM grids were used for TEM characterisations. HR-TEM images were recorded in Thermo Fisher Scientific Talos F200X system operated at 80 KV. HAADF STEM and EDS measurements were performed in aberration correction FEI Titan G2 ChemiSTEM system operated at 200 KV and 60 pA beam current. Grazing incidence X-ray scattering measurements (GIWAXS) were carried out on the XMaS [2] beamline at the European Synchrotron Radiation Facility (ESRF). GIWAXS was performed using a monochromatic X-ray beam energy of 10.0 KeV (λ = 1.24 Å) and scattering patterns were recorded on a Pilatus3 1M detector (Dectris) with a sample-to-detector distance of 282 mm, calibrated using a [3] silver behenate standard. Alignment was performed via three iterative height (z) and rocking (Ω) scans, before the grazing incidence angle was set to $\Omega = 0.16$ °. A two-dimensional image was recorded with an exposure time of 60 s. An angle scan was conducted between grazing incidence angles of $\Omega = 0.10$ ° and $\Omega = 0.30$ ° in 0.02 ° increments, with each two-dimensional image recorded with an exposure time of 60 s. The two-dimensional scattering patterns were masked to remove the substrate horizon, detector module gaps and the beam-stop before being reduced to one-dimensional scattering data via radial integration. This was done in python using the PyGIX library.[4]

4. DFT methodology

Density functional theory was performed for sodium ion adsorption studies. Becky's three-parameter exchange function, combined with Lee-Yang-Parr correlation functional (B3LYP) method with 6-31g(d) basis set, and Los Alamos National Laboratory 2 Double-Zeta method (LANL2DZ) was utilized, where CuMOF fragment optimization and CuMOF one-ring frequency were calculated in Gaussian16

software package. The free energy (ΔG) of adsorption and desorption was expected to be identical in this calculation, as we assume no other chemical species are affecting the sorption behaviour. This is shown in the following equation:

$$\Delta G = G_{Na^{+}CuMOF} - G_{Na^{+}} - G_{CuMOF}$$

Using the output file from Gaussian16 software package, electrostatic potential (ESP) topology analysis was calculated by Multiwfn Version 3.8[5-6] and visualized by VMD (Visual Molecular Dynamics).[3]

5. XPS Experimental Section

X-ray Photoelectron Spectroscopy (XPS) was performed using an Axis Supra+ spectrometer (Kratos Analytical, Manchester, United Kingdom) using monochromated Al Kα radiation (1486.6 eV, 20 mA emission at 300 W, spot size 1 mm) [or Ag Lα (2984.2 eV, 30 mA emission at 450 W, spot size 1mm)] with a base vacuum pressure of ~5×10^{-9} mbar. Charge neutralisation was achieved using a filament. The binding energy scale calibration was performed using C-C in the C 1s photoelectron peak at 284.8 eV.[7] Analysis and curve fitting were performed using Voigt-approximation peaks using CasaXPS.[8]

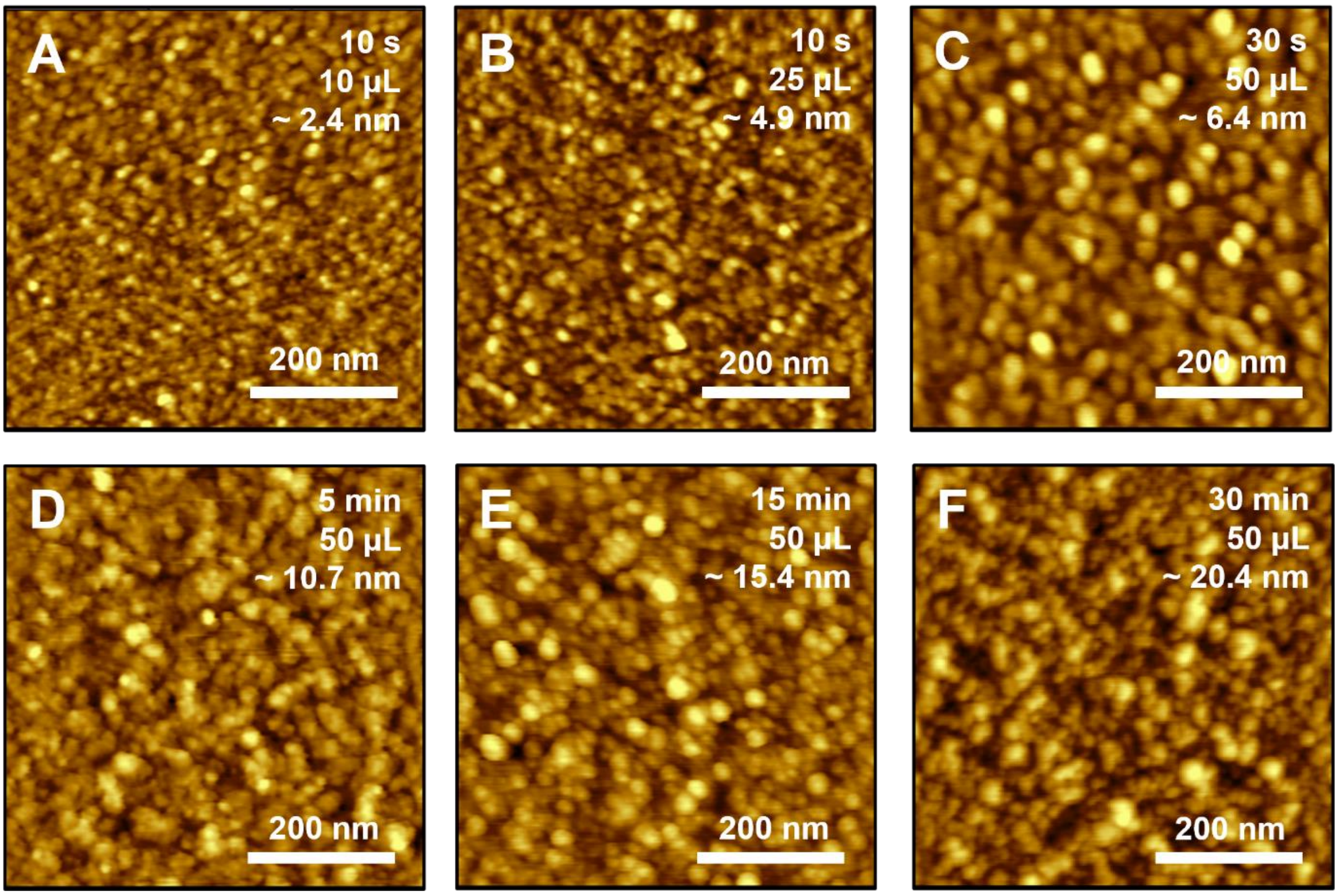

Fig. S5. AFM surface images for CuMOFs of different thicknesses. These images were used for roughness calculation. The time of the reaction and thickness of the film are indicated on each picture, along with the volume of the ligand.

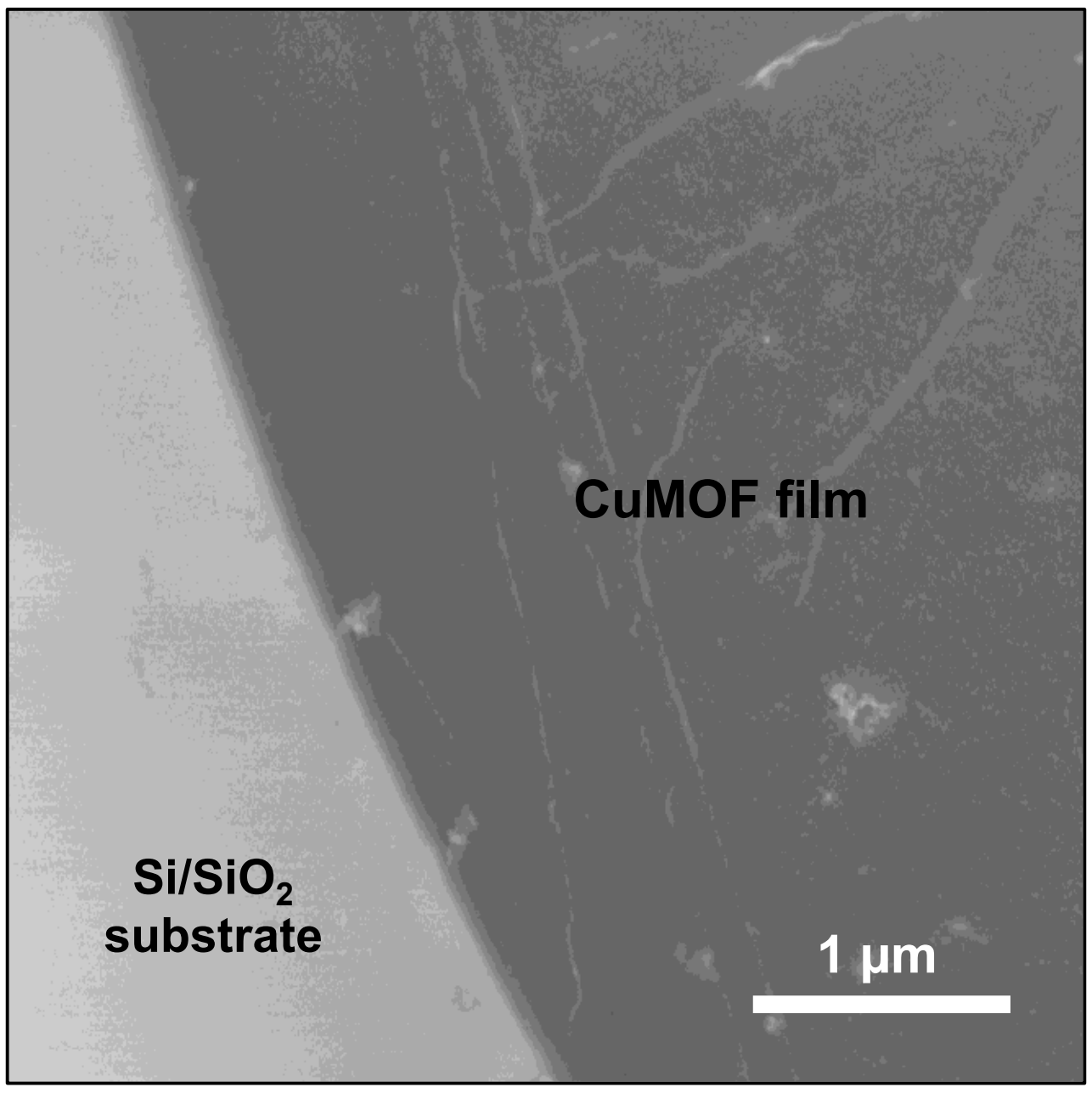

Fig. S6: FESEM image of CuMOF. The CuMOF film scooped onto a Si/SiO_2 substrate.

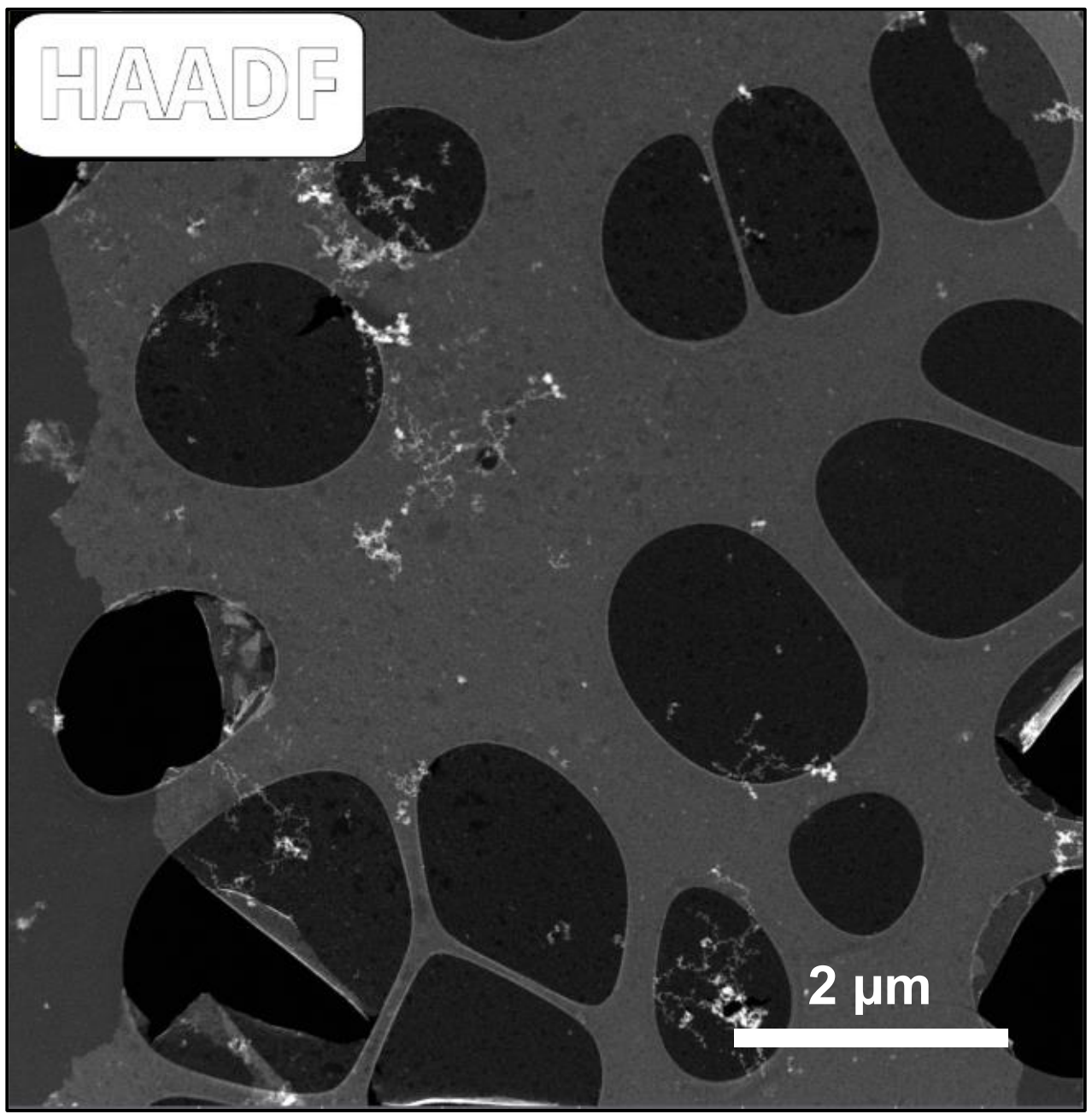

Fig. S7. HAADF-STEM image of CuMOF. CuMOF membrane scooped onto a TEM gold grid with a holey carbon support membrane.

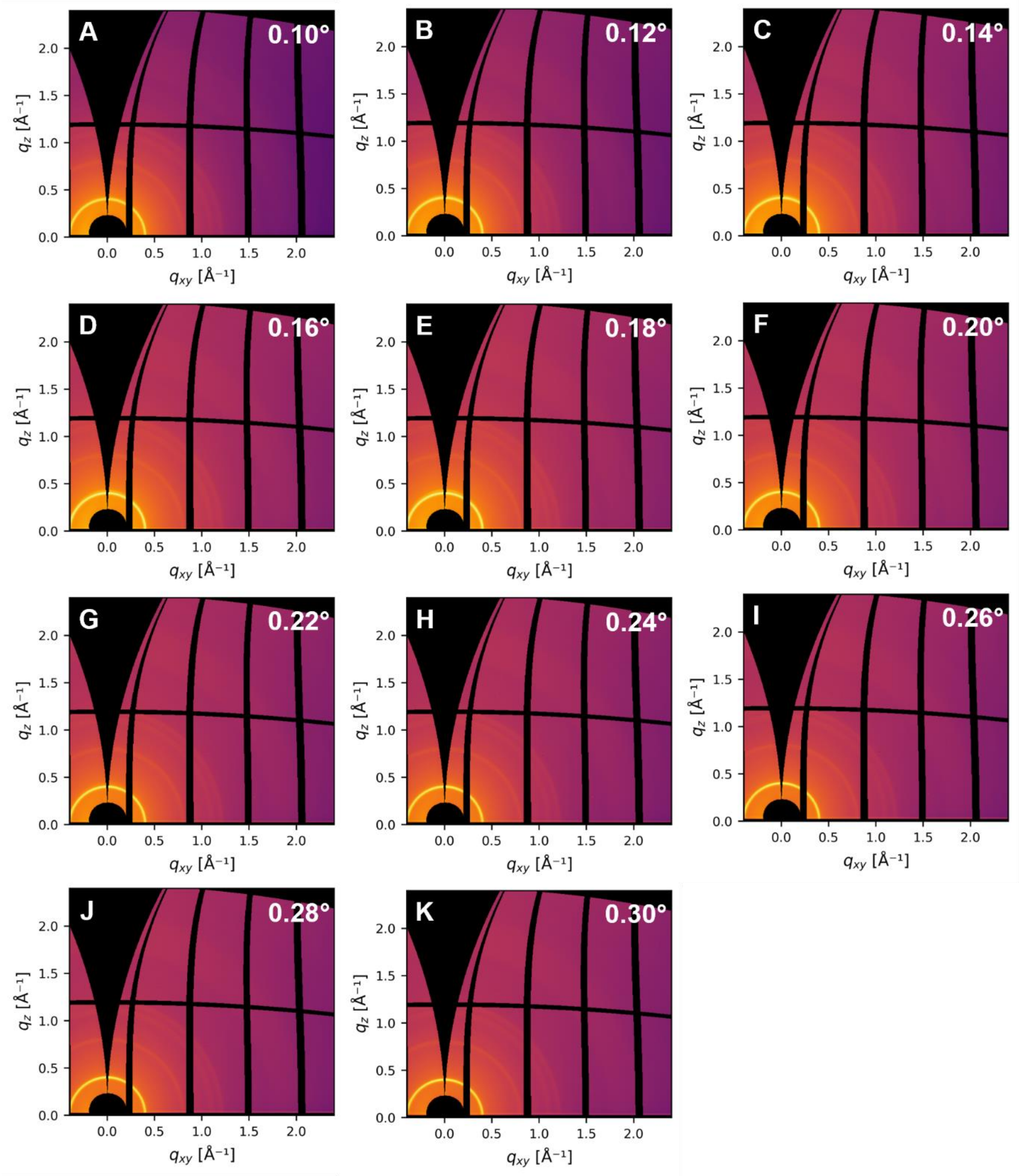

Fig. S8. GIWAXS patterns of CuMOF at different grazing incidences. 2D-GIWAXS data collected at different grazing incidence angles from 0.1° to 0.3° (**A-K**) to probe both the surface and bulk of the film.

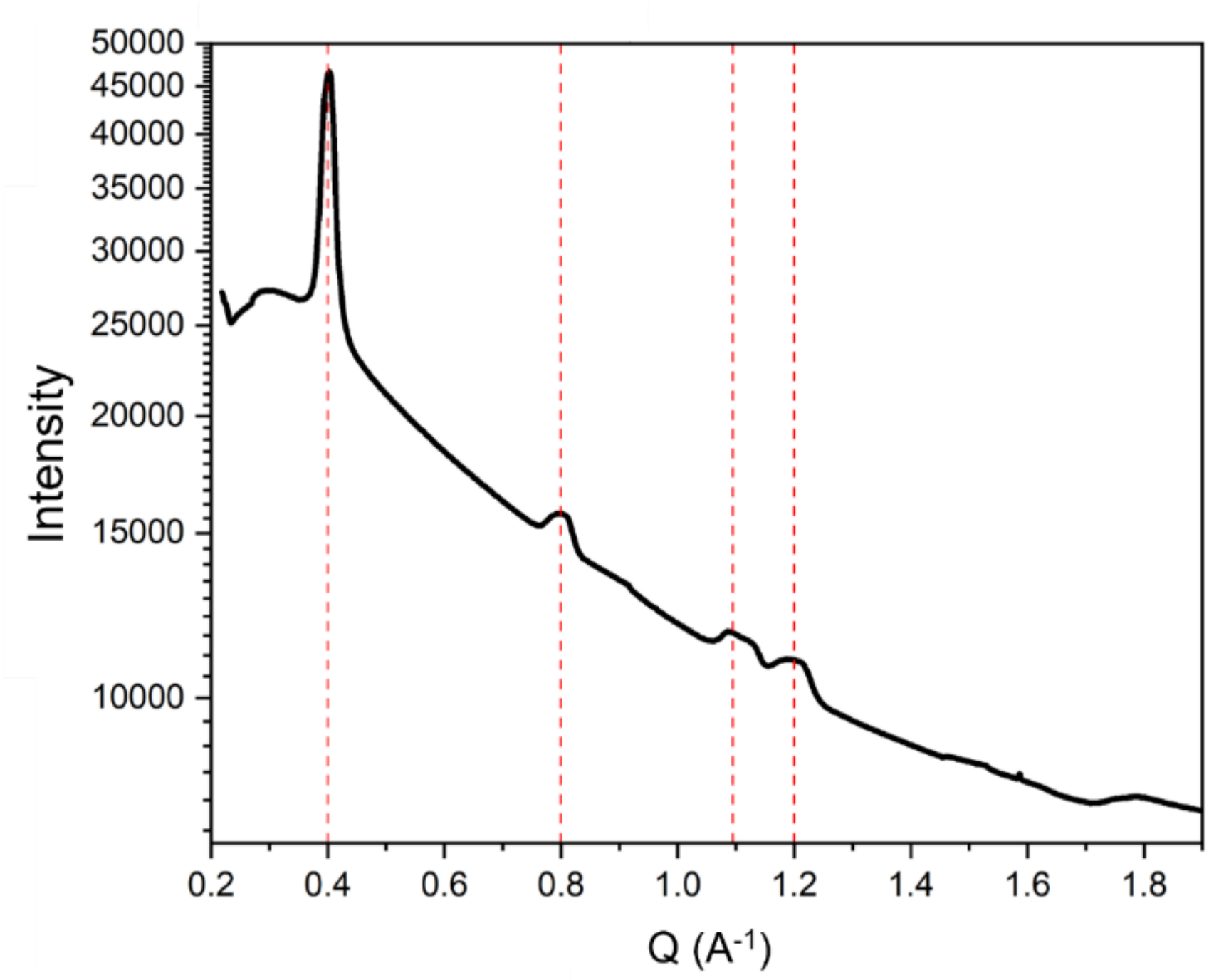

Fig. S9. GIWAXS profile data of CuMOF film. The data were from a 165 nm thick CuMOF film on a SiN_x substrate.

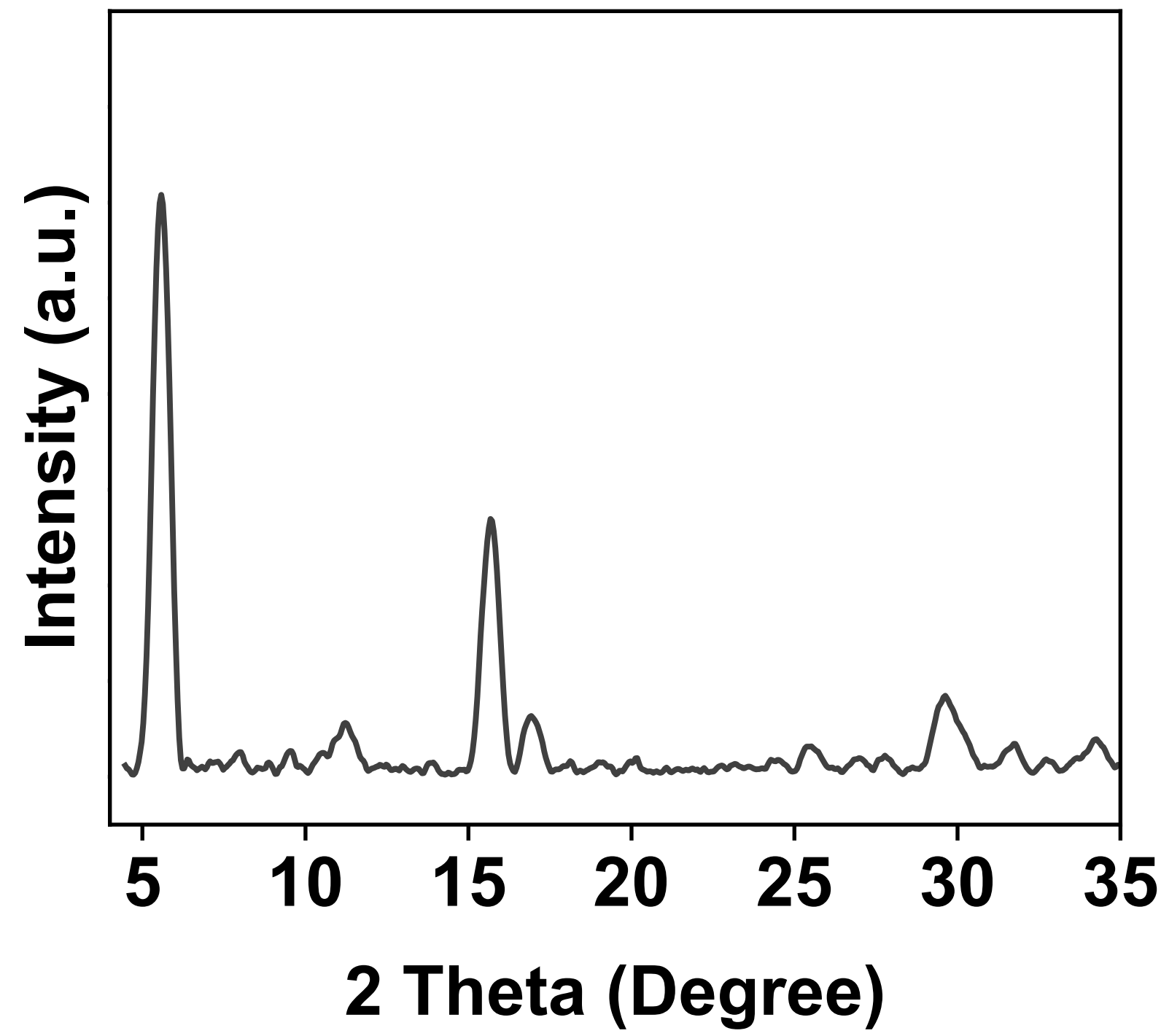

Fig. S10. GIXRD data of CuMOF film. A CuMOF film (165 nm thick) scooped onto a SiN_x substrate.

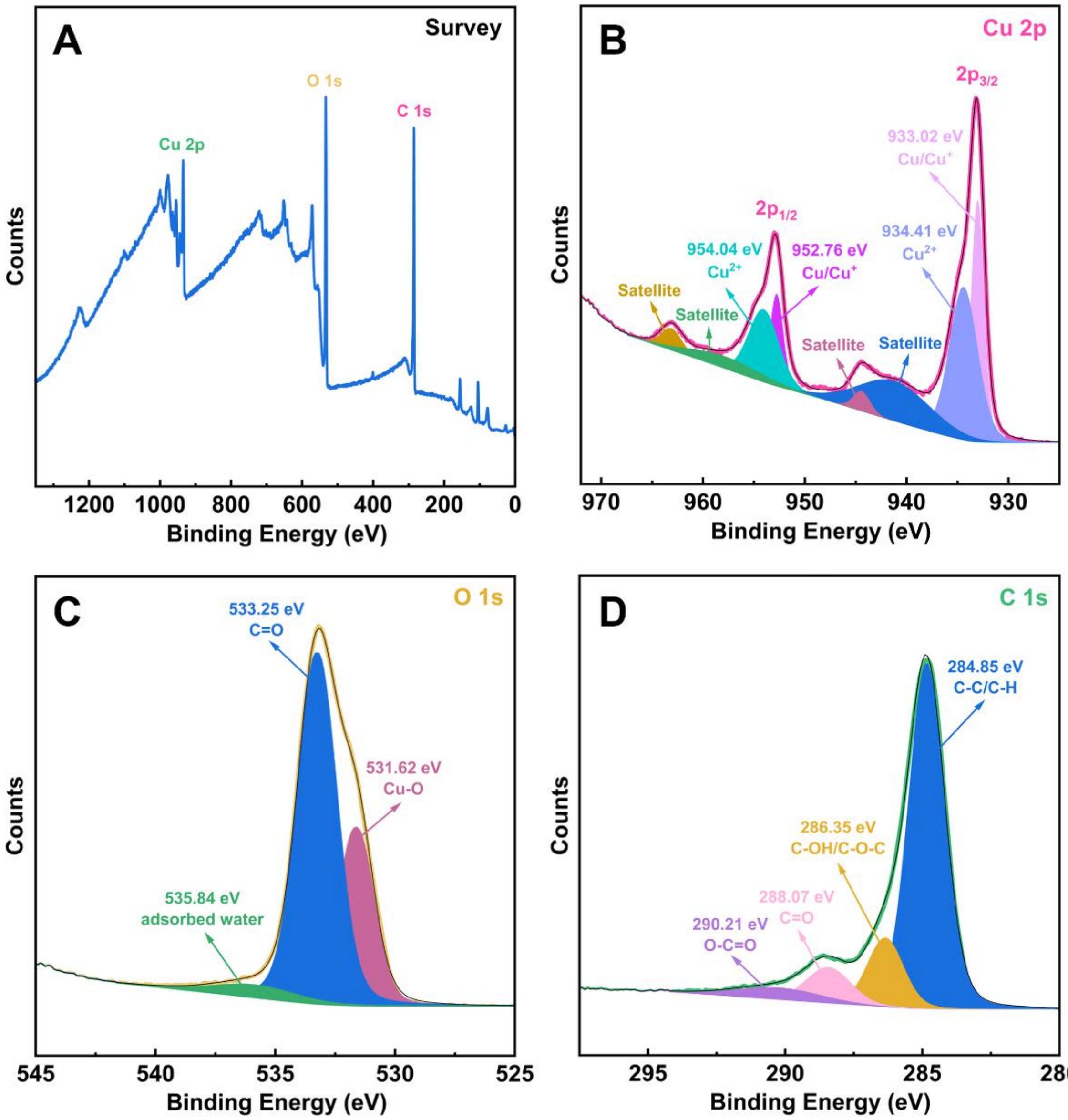

Fig S11. XPS characterization of the Cu–HHTP thin film. (**A**) Survey spectrum showing only Cu, O, and C signals, confirming high chemical purity. (**B**) High-resolution Cu 2p spectrum exhibiting characteristic Cu^{2+} peaks with shake-up satellites. (**C**) O 1s spectrum showing components associated with Cu–O bonding and phenolic/quinone oxygen in the HHTP ligand. (**D**) C 1s spectrum featuring aromatic and oxygenated carbon species consistent with the intact HHTP framework.

6. Ion transport measurements

A custom-made electrochemical setup, consisting of two electrolyte chambers made from polyether ether ketone (PEEK), was used to perform all ion transport measurements. The SiN_X chip containing the MOF nanoporous film was placed between the two reservoirs, with O-rings between each part to ensure leak-tight sealing. For all ion transport measurements, 2 mL of electrolyte was used in each experiment, with the concentration depending on the intended experiment. Custom-made Ag/AgCl electrodes were immersed in each reservoir, and electrochemical measurements were recorded using a Keithley 2636B SourceMeter with LabVIEW software. Before measurements with electrolytes, the channels in the electrochemical setup were thoroughly washed with deionised (DI) water, followed by a 10% isopropyl alcohol/water mixture, and then rinsed again with DI water in a repetitive manner to ensure cleanliness.

7. Surface-area dependent conductance of CuMOF devices

The increase in the surface area increases the *G* of the CuMOF devices (Fig. S12B) as expected. However, for a given thickness, the *G* per area remains similar. Fig. S12B inset compares the *G* per area for CuMOF devices with different surface areas, but of similar thickness of 15 nm.

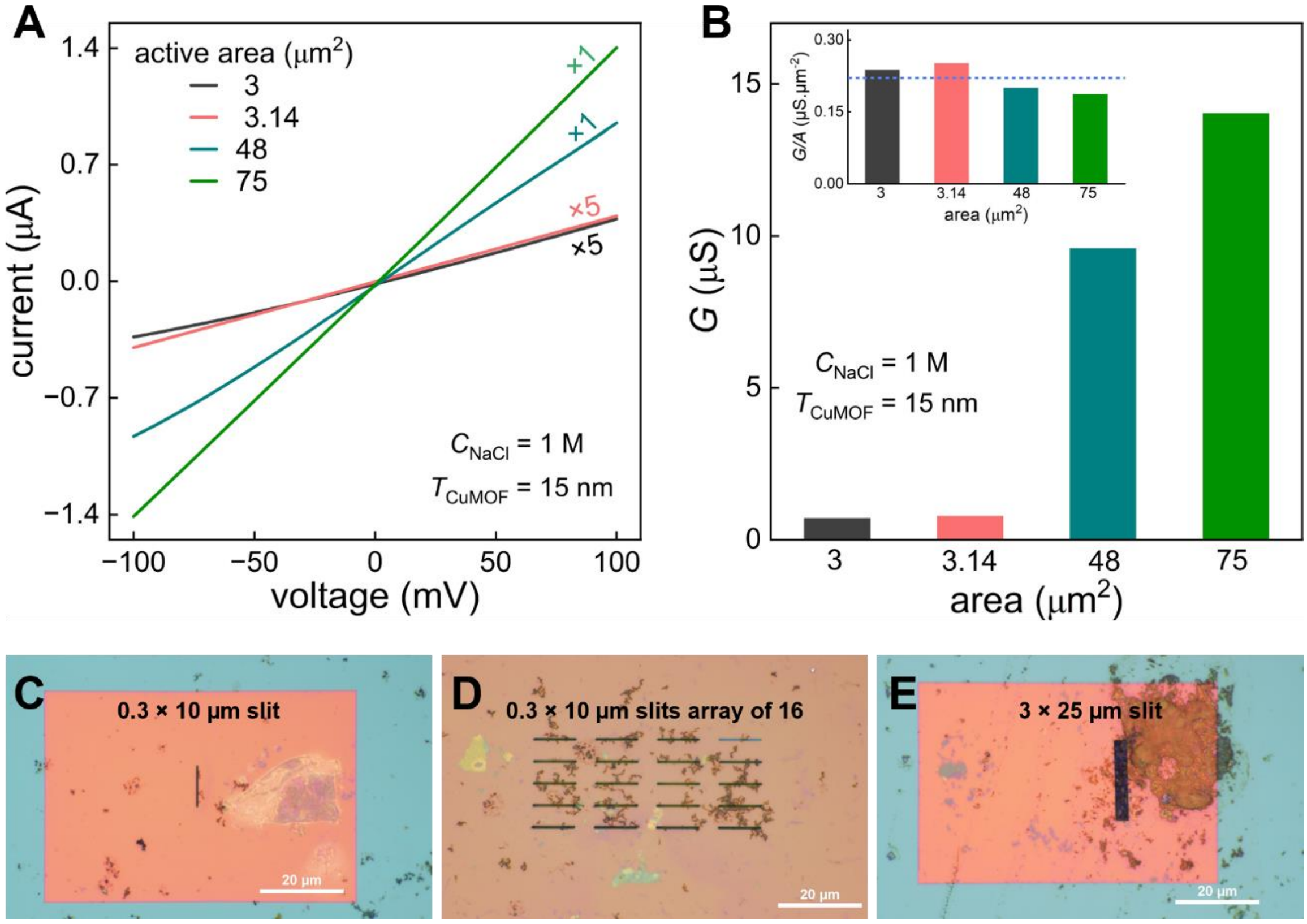

Fig. S12: Surface area dependent *G* of CuMOF devices with T_{CuMOF} = 15 nm. (**A**) Comparison of the *I-V*s of devices with different surface areas. The *I-V* curves were multiplied by different factors (indicated for each curve with same colour code) for better visualisation. (**B**) Bar diagram comparing the *G* of CuMOF devices in (**A**) with different surface areas. Inset compares the normalized *G* with the effective surface area of the devices, with the mean value represented by the blue dotted line. 1 M NaCl is used as electrolyte in this figure. Optical images of CuMOF devices with different exposed areas (**C**) 3 μm^2, (**D**) 48 μm^2 and (**E**) 75 μm^2 (images are recorded after ion transport measurements).

8. Long-term and chemical stability of CuMOF devices

The long-term immersion stability of the CuMOF device (thickness, 15 nm) was investigated in an electrolyte over 2500 *I-V* cycles. Initially, the device was subjected to 1000 *I-V* cycles in 1 mM NaCl over a period of approximately 25 hours (Fig. S13A), followed by resting in the same electrolyte until a total immersion time of 114 hours. Subsequently, the electrolyte was replaced with 100 mM NaCl, and an additional 1500 *I-V* cycles were recorded over 56 hours (Fig. S13A). In total, the device underwent 2500 *I-V* cycles with a cumulative immersion time of ~170 hours. The conductance (*G*) evolution and the corresponding *I-V* characteristics are shown in (Fig. S13C), indicating the robustness of the device.

Additionally, we evaluated the chemical robustness of another device (15 nm thickness) by recording it's *I-V*s in 100 mM NaCl solutions across varying pH conditions (Fig. S13D inset). A decrease in pH resulted in a modest reduction in *G* (Fig. S13D), likely due to the protonation of negatively charged surface groups within the channel.[9] Conversely, raising the pH produced a slight increase in *G*, which can be attributed to an increase in effective surface-charge-density. The pH was not extended beyond 9

due to the known instability of the SiN_X substrate under strongly alkaline conditions. These observations indicate that ion transport in the CuMOF device is predominantly governed by surface-charge-regulated conduction mechanisms and confirm that the device maintains chemical stability within the investigated pH range.

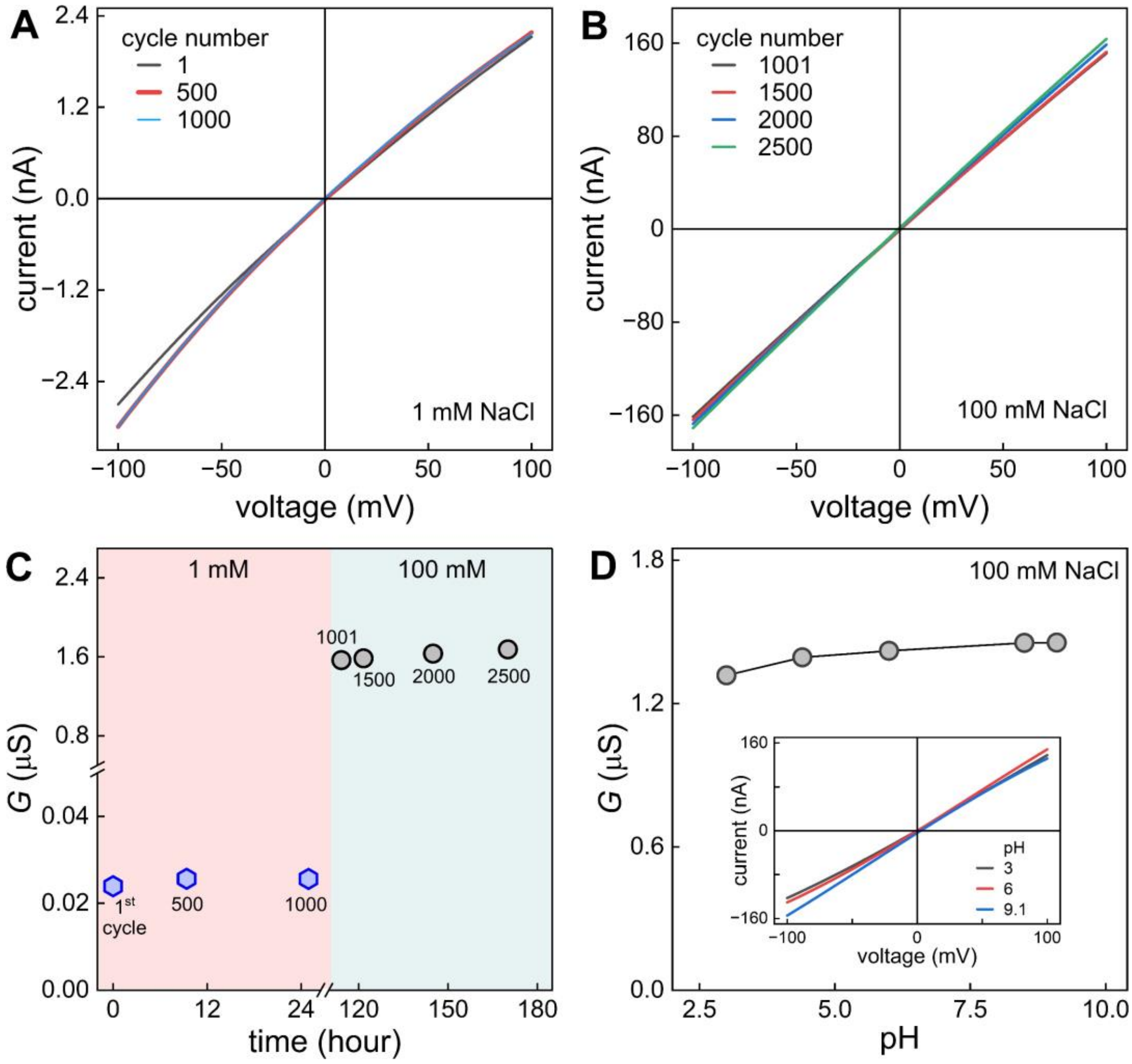

Fig. S13: Stability of CuMOF devices. Comparison of *I-V* curves through a device with (**A**) 1 mM NaCl and (**B**) 100 mM NaCl as electrolytes. (**C**) Long-term immersion stability (conductance, *G*) of the device over 2500 *I-V* cycles; first 1000 cycles in 1 mM NaCl (~25 hours and total immersion 114 hours), followed by 1500 cycles in 100 mM NaCl (56 h), reaching a total immersion time of ~170 hours. (**D**) pH dependent *G* of a device in 100 mM NaCl. Inset in (**D**) compares the *I-V* curves at pH 3, 6, and 9.1. Two different devices (each with T_{CuMOF} = 15 nm) were used in this figure: (**A**), (**B**) and (**C**) are from one device and (**D**) from a different device.

9. Nanofluidic ion transport through CuMOF devices

Table S1: Parameters for different NaCl solutions for calculating transference number.[10]

C_H (M)	γ_H	C_L (M)	γ_L	E_R (mV)	E_T (mV)	E_M (mV)	t_{Na^+}	t_{Cl^-}
10^{-3}	0.965	10^{-4}	1	58	102	44	0.88	0.12
10^{-2}	0.903	10^{-3}	0.965	57	106	49	0.92	0.08
10^{-1}	0.779	10^{-2}	0.903	55	92	37	0.83	0.17
1	0.657	10^{-1}	0.779	55	66	11	0.60	0.40

Table S2: Comparison of the bulk transference of ions in different electrolytes.

Electrolyte	t_{cation}	t_{anion}
**KCl**	0.49	0.51
**NaCl**	0.4	0.6
**LiCl**	0.34	0.66

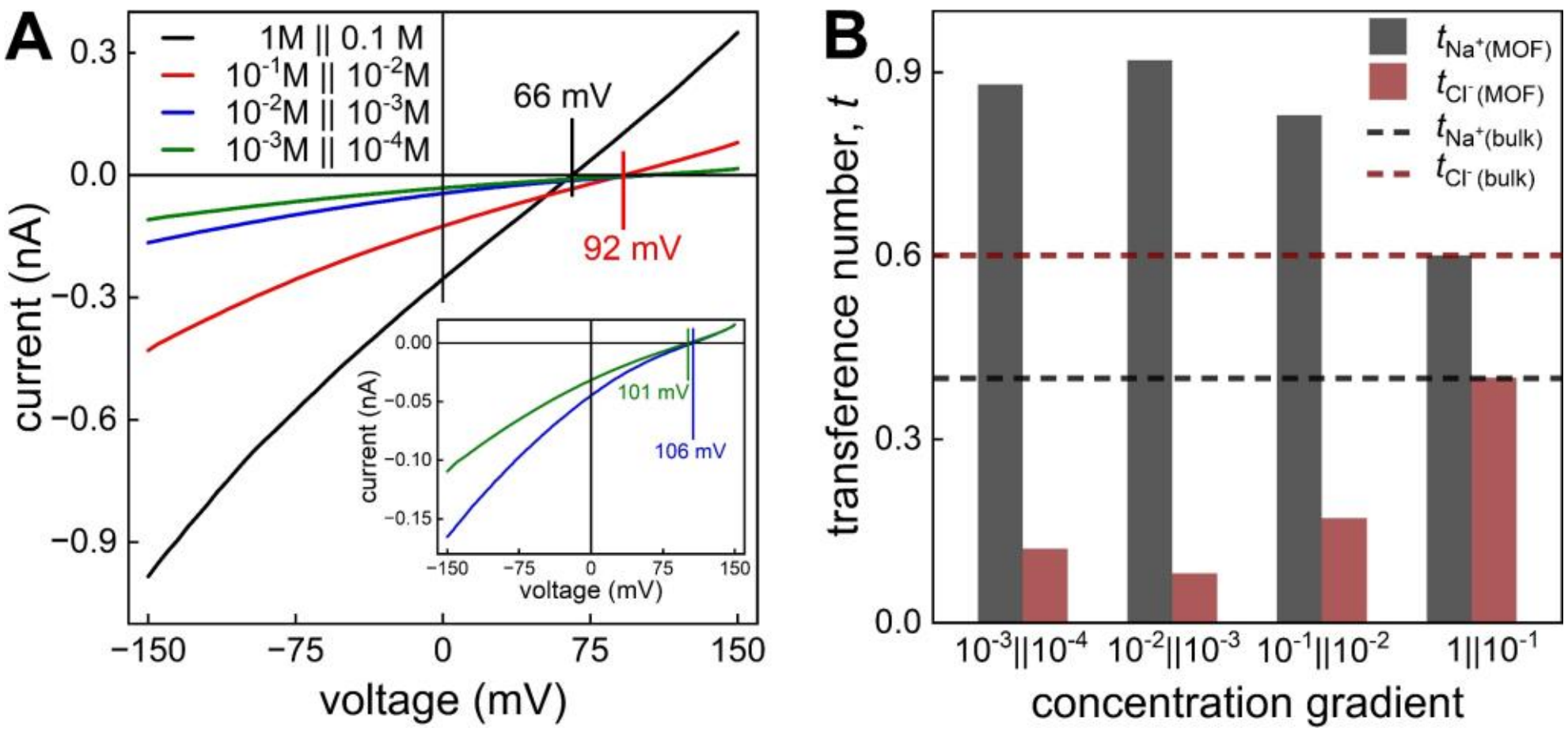

Fig. S14: Drift diffusion of CuMOF device with T_{CuMOF} = 20 nm. (**A**) *I-V* curves for a CuMOF device under ΔC = 10 across the device, with different C_{NaCl}. The voltage-axis intercept represents the total potential (E_T) generated by ΔC, which includes the redox potential (E_R) from the Ag/AgCl electrode. The inset shows zoomed-in *I-V* curves for 10^{-2} M || 10^{-3} M and 10^{-3} M || 10^{-4} M gradients for better clarity. In (**A**), the C_{NaCl} in the reservoir containing the counter electrode (*CE*) is maintained at a higher concentration than that in the reservoir with the working electrode (*WE*). (**B**) Transference numbers (*t*) of Na^+ and Cl^- ions of a CuMOF device at different concentrations under ΔC = 10.

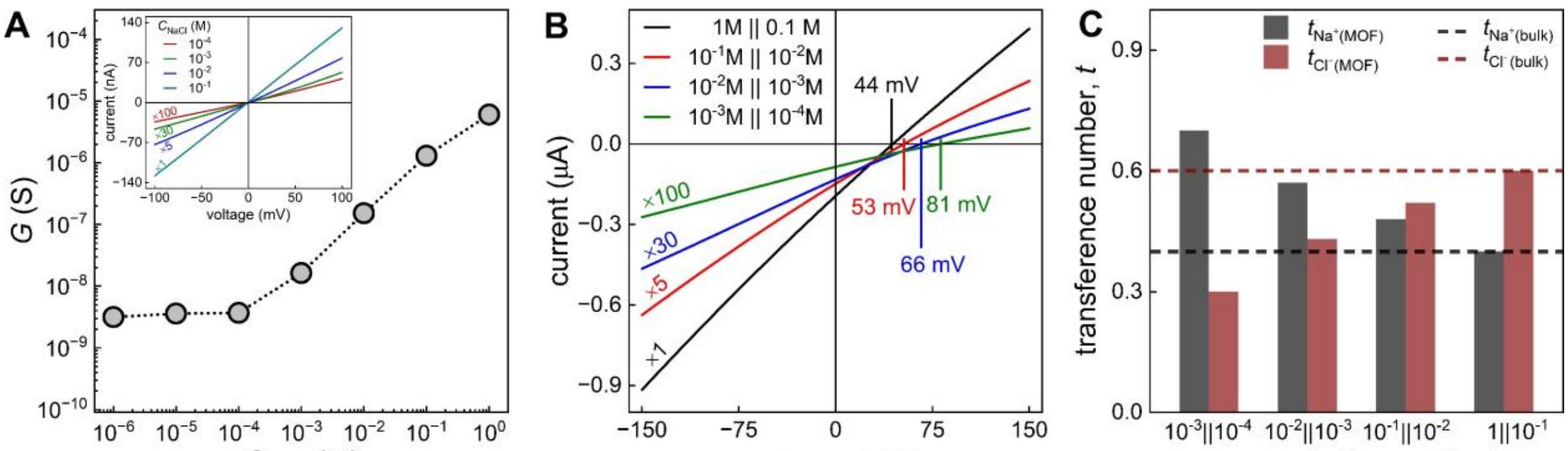

Fig. S15: Nanofluidic transport of CuMOF device with T_{CuMOF} = 2.4 nm. (**A**) *G* of a CuMOF device as a function of NaCl concentration (C_{NaCl}). The inset shows *I-V* curves for the MOF device at different C_{NaCl} (**B**) *I-V* curves for a CuMOF device under ΔC = 10 across the device, with different C_{NaCl}. The voltage-axis intercept represents the total potential (E_T) generated by ΔC, which includes the redox potential (E_R) from the Ag/AgCl electrode. In (**B**), the C_{NaCl} in the reservoir containing the counter electrode (*CE*) is maintained at a higher concentration than that in the reservoir with the working electrode (*WE*). (**C**) Transference numbers (*t*) of Na^+ and Cl^- ions of a CuMOF device at different concentrations under ΔC = 10. The curves in **(B)** and the inset of (**A**) are multiplied as indicated, for better comparison.

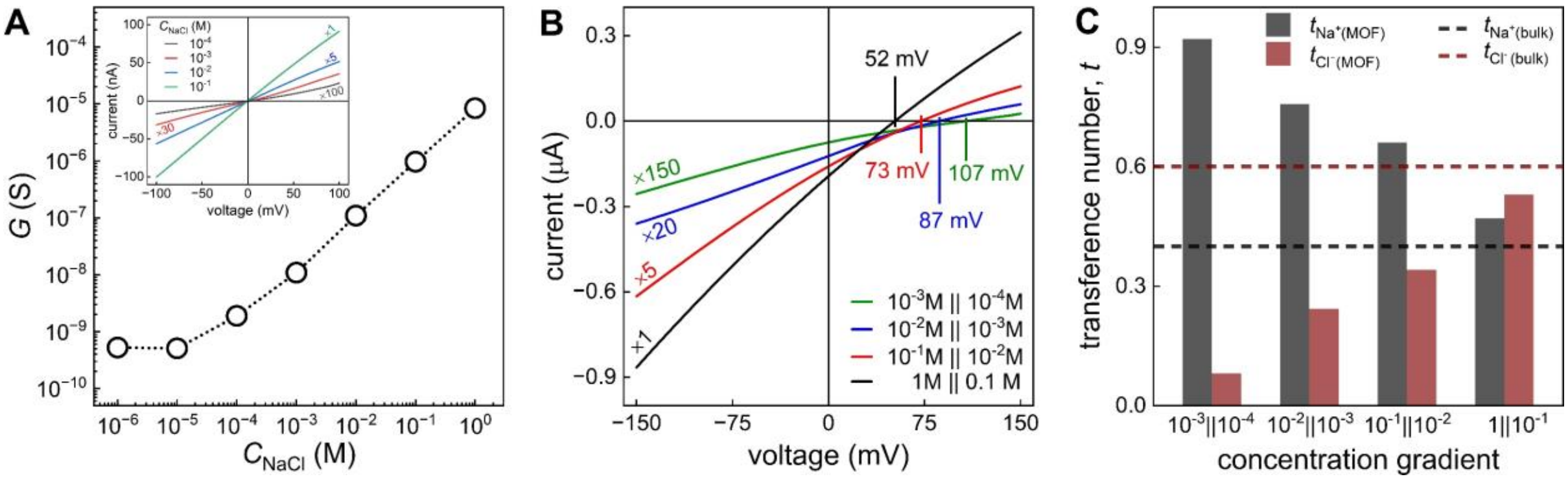

Fig. S16: Nanofluidic transport of CuMOF device with T_{CuMOF} = 9.5 nm. (**A**) *G* of a CuMOF device as a function of NaCl concentration (C_{NaCl}). The inset shows *I-V* curves for the MOF device at different C_{NaCl}. (**B**) *I-V* curves for a CuMOF device under ΔC = 10 across the device, with different C_{NaCl}. The voltage-axis intercept represents the total potential (E_T) generated by ΔC, which includes the redox potential (E_R) from the Ag/AgCl electrode. In (**B**), the C_{NaCl} in the reservoir containing the counter electrode (*CE*) is maintained at a higher concentration than that in the reservoir with the working electrode (*WE*). (**C**) Transference numbers (*t*) of Na^+ and Cl^- ions of a CuMOF device at different concentrations under ΔC = 10. The curves in (**B**) and the inset of (**A**) are multiplied as indicated, for better comparison.

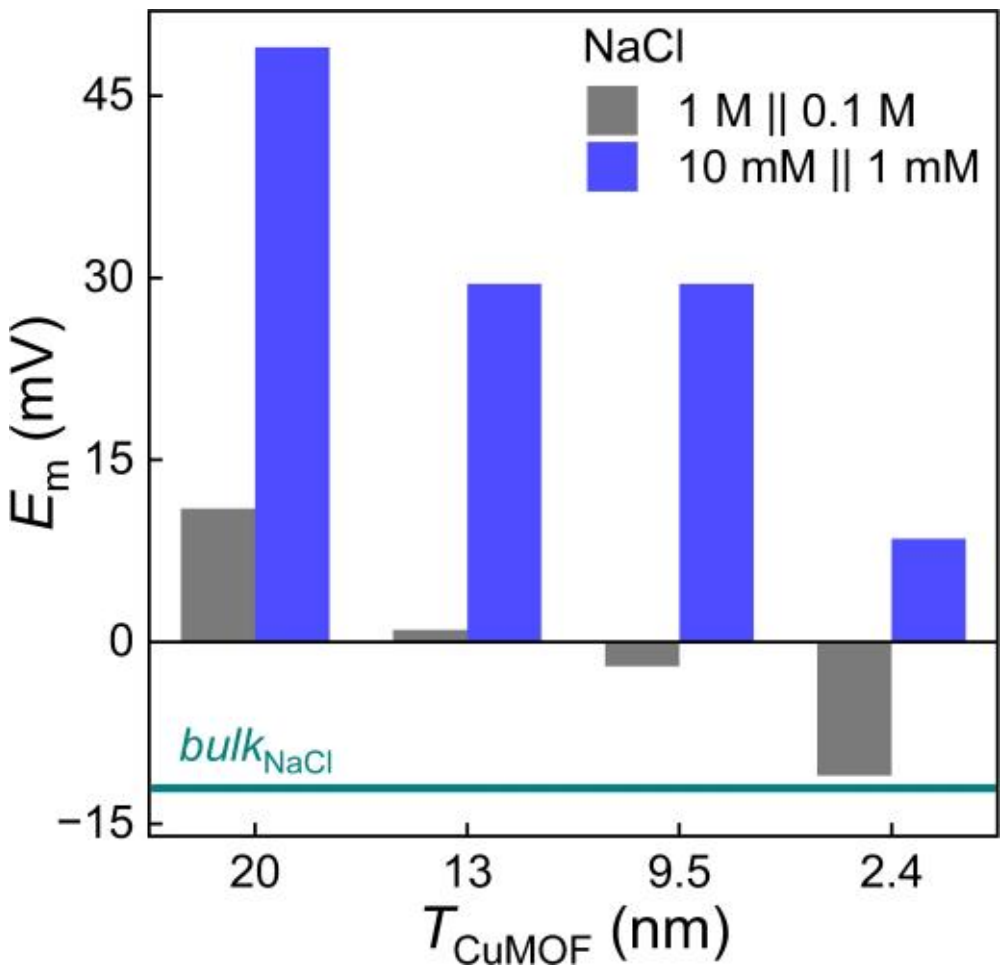

Fig. S17: T_{CuMOF} dependent E_M under a ΔC =10 with C_{feed} = 1 M and $C_{permeate}$ = 0.1M (black bars) and C_{feed} = 10 mM and $C_{permeate}$ = 1 mM (blue bars).

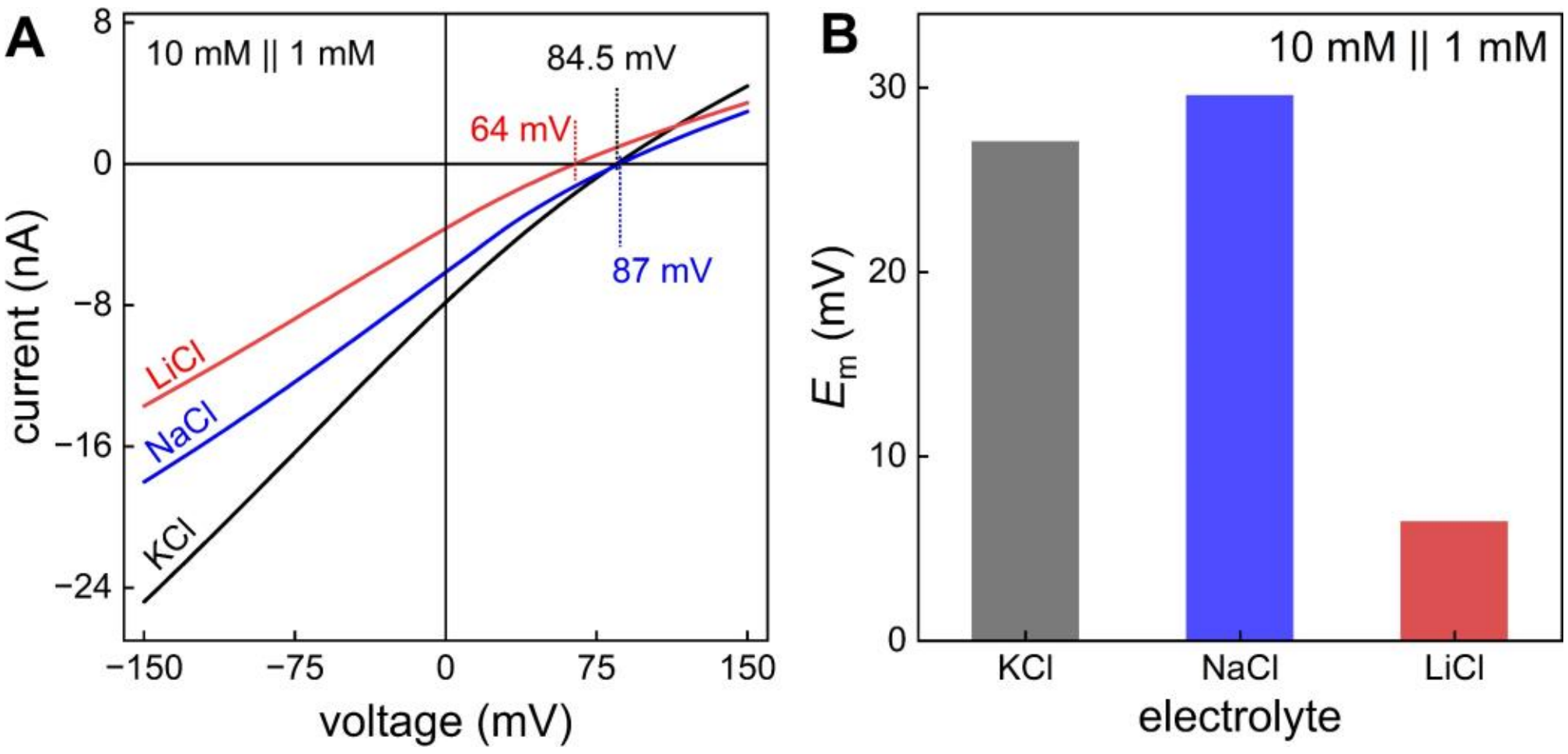

Fig. S18: Electrolyte dependent E_m. Comparison of the (**A**) *I-Vs* and (**B**) E_m for KCl, NaCl and LiCl, each under a ΔC =10 with C_{feed} = 10 mM and $C_{permeate}$ = 1 mM. The zero-current potentials in (**A**) have the electrode contributions. T_{CuMOF} = 9.5 nm.

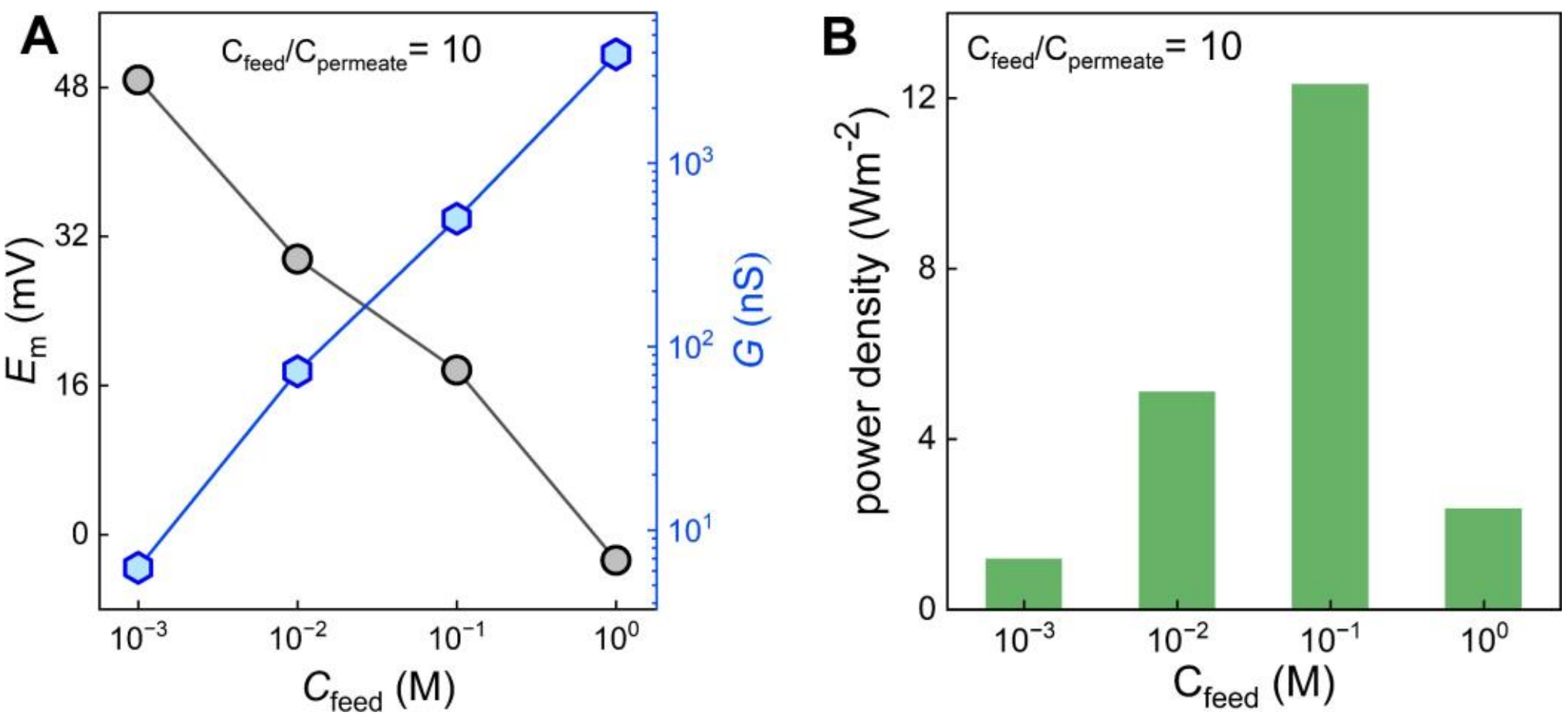

Fig. S19: Osmotic energy. Comparison of the (**A**) E_m and G and (**B**) Osmotic power density at different NaCl concentrations under a ΔC = 10 (the corresponding *I-V*s are in Fig. S16B). T_{CuMOF} = 9.5 nm

10. Ionic memory in CuMOF devices

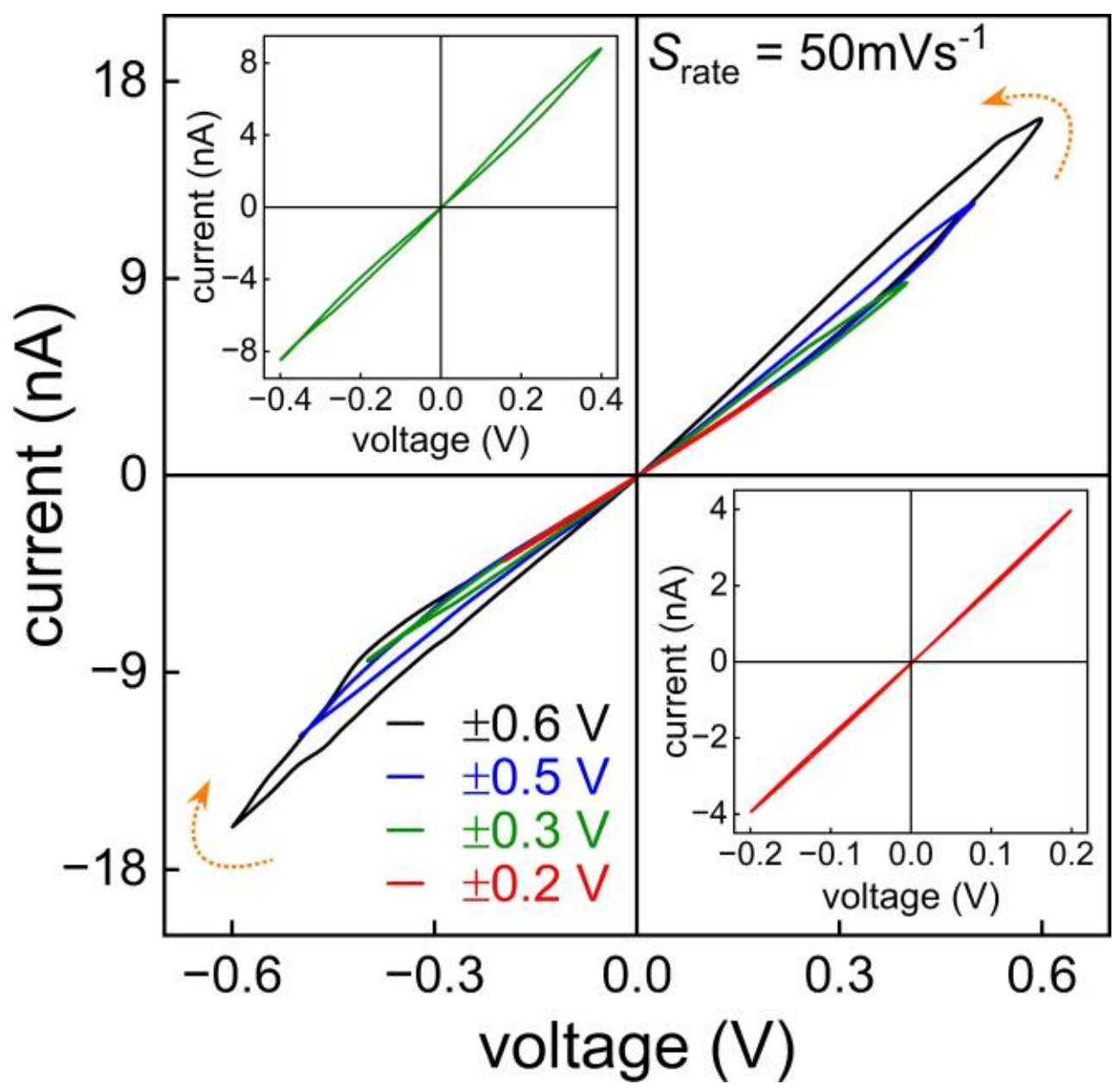

Fig. S20: Voltage range dependent *I-V* loops of CuMOF device (T_{CuMOF} = 20 nm) using 0.5M NaCl as electrolyte. The memristive curves are self-crossing (bipolar). The scan rate is fixed at 50 mV.s^{-1} for each loop.

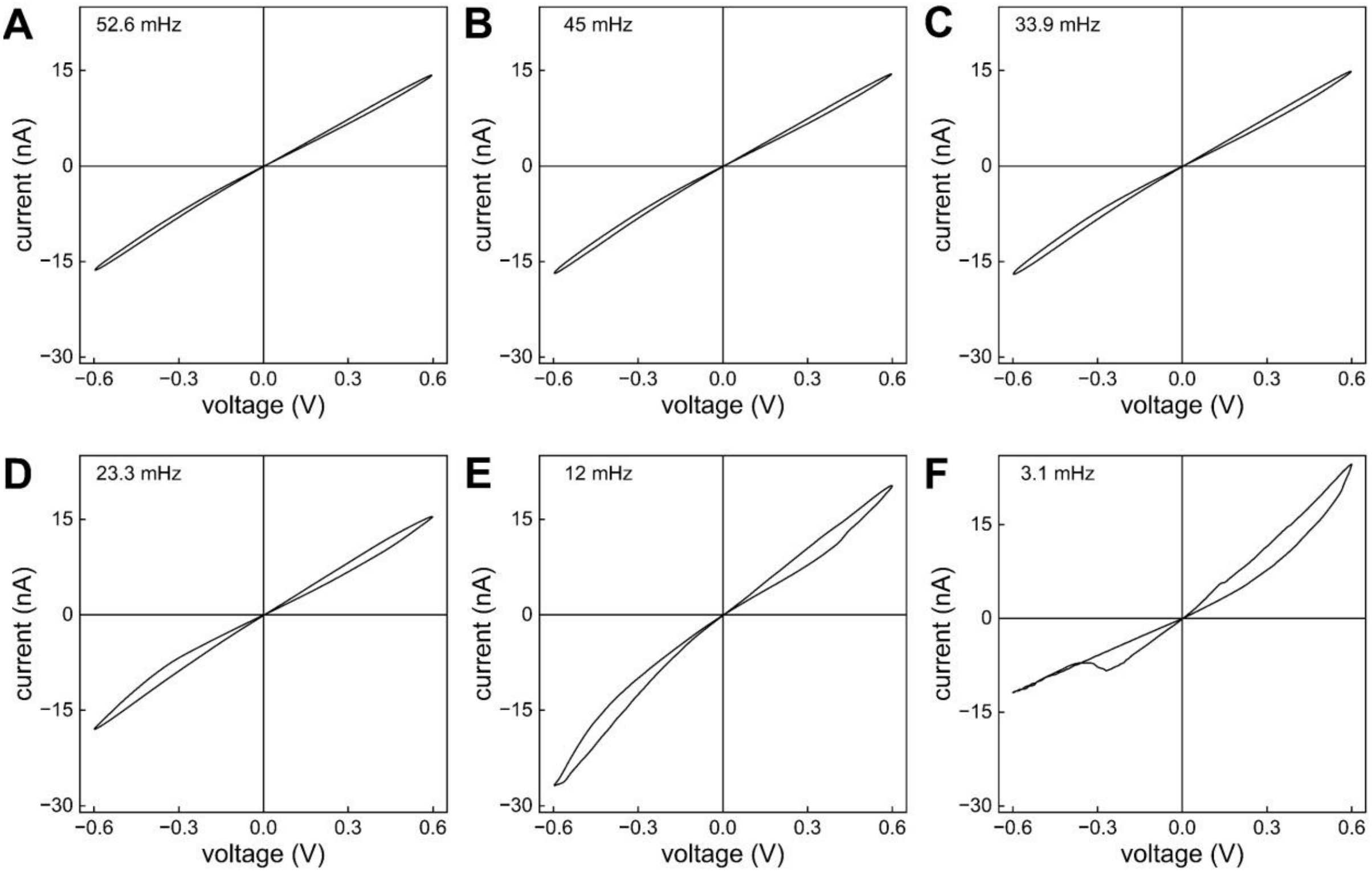

Fig. S21: Frequence dependent *I-V* loops: Self-crossing memristive loops at (**A**) 52.6 mHz, (**B**) 45 mHz, (**C**) 33.9 mHz, (**D**) 23.3 mHz, (**E**) 12 mHz and (**F**) 3.1 mHz of CuMOF device (T_{CuMOF} = 20 nm) using 0.5M NaCl as electrolyte.

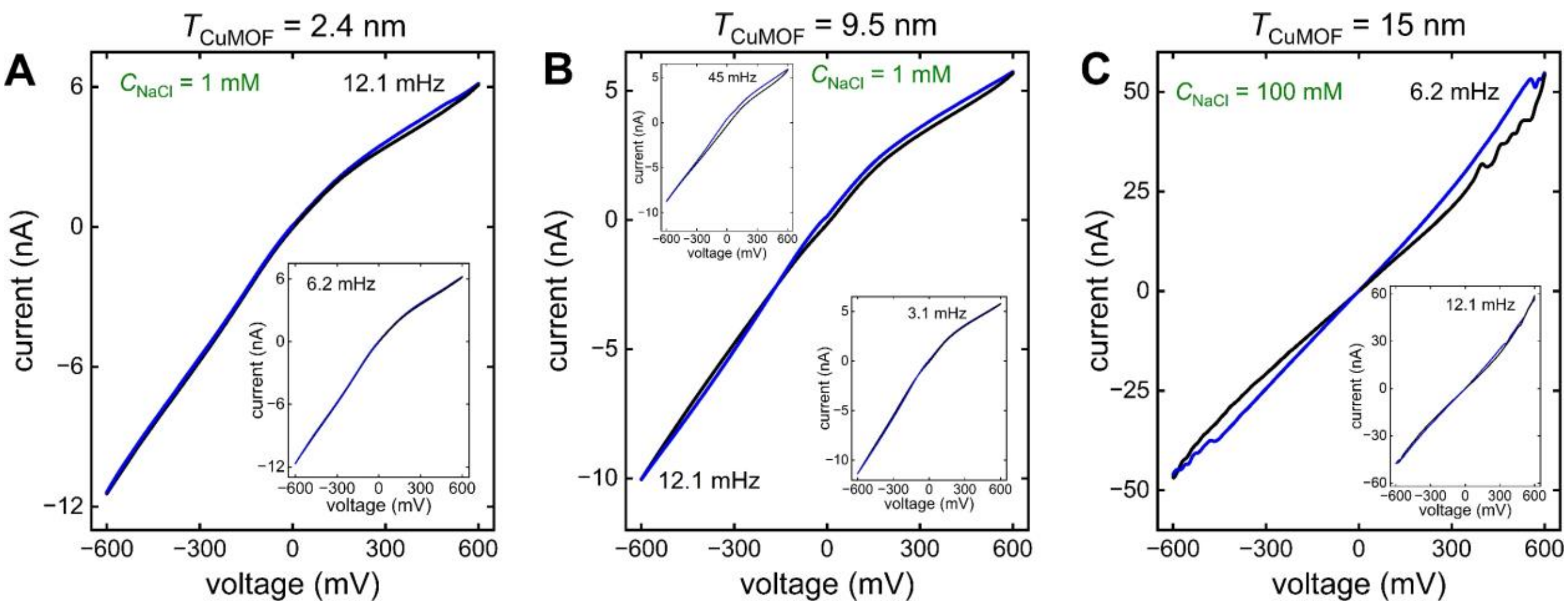

Fig. S22. Thickness-dependent *I-V* loops of CuMOF membranes. CuMOF membrane with T_{CuMOF} (**A**) 2.4 nm measured in 1 mM NaCl at 12.1 mHz (inset at 6.2 mHz), (**B**) 9.5 nm measured in 1 mM NaCl at 12.1 mHz (insets at 45 mHz and 3.1 mHz) and (**C**) 15 nm measured in 100 mM NaCl at 6.2 mHz (inset at 12.1 mHz).

The T_{CuMOF} plays a critical role in governing the memristive response of the CuMOF devices. We systematically investigated the *I-V* loops of CuMOF film with T_{CuMOF} = 2.4, 9.5, 15, and 20 nm under varying NaCl concentrations (1 mM to 1 M) and frequencies. For T_{CuMOF} = 2.4 nm, negligible hysteresis was observed across the entire concentration range and at all tested frequencies. Only a very small loop area appeared at 1 mM NaCl with 12.1 mHz (Fig. S22A), indicating the absence of a pronounced memristive effect. Increasing the thickness to T_{CuMOF} = 9.5 nm similarly resulted in minimal hysteresis, with only a slight loop area detected at 1 mM NaCl at 12.1 and 45 mHz (Fig. S22B). These results suggest that ultrathin membranes do not show memory behaviour. When the thickness was further increased to 15 nm, a more evident hysteresis loop emerged, particularly at 100 mM NaCl and 6.2 mHz

(Fig. S22C). However, the loop area decreased upon increasing the frequency to 12.1 mHz (Fig. S22C, inset) or altering the concentration to 10 mM or 1 M. In addition, the hysteresis response was not reproducible over repeated measurements (Fig. S23A), indicating an unstable and non-retentive memory effect at this intermediate thickness. A stable and reproducible memristive behavior was achieved for T_{CuMOF} = 20 nm; supported by the frequency-dependent *I-V* (Fig. S21), quantitative loop area analysis (Fig. 4C), consecutive positive and negative voltage cycling (Fig. 4D), memory retention (Fig. S24), potentiation and depression characteristics (Fig. 4E), and three consecutive repetitive cycles (Fig. S23B). These confirm that increased membrane thickness enables stable ionic modulation and reliable memristive functionality in the CuMOF devices.

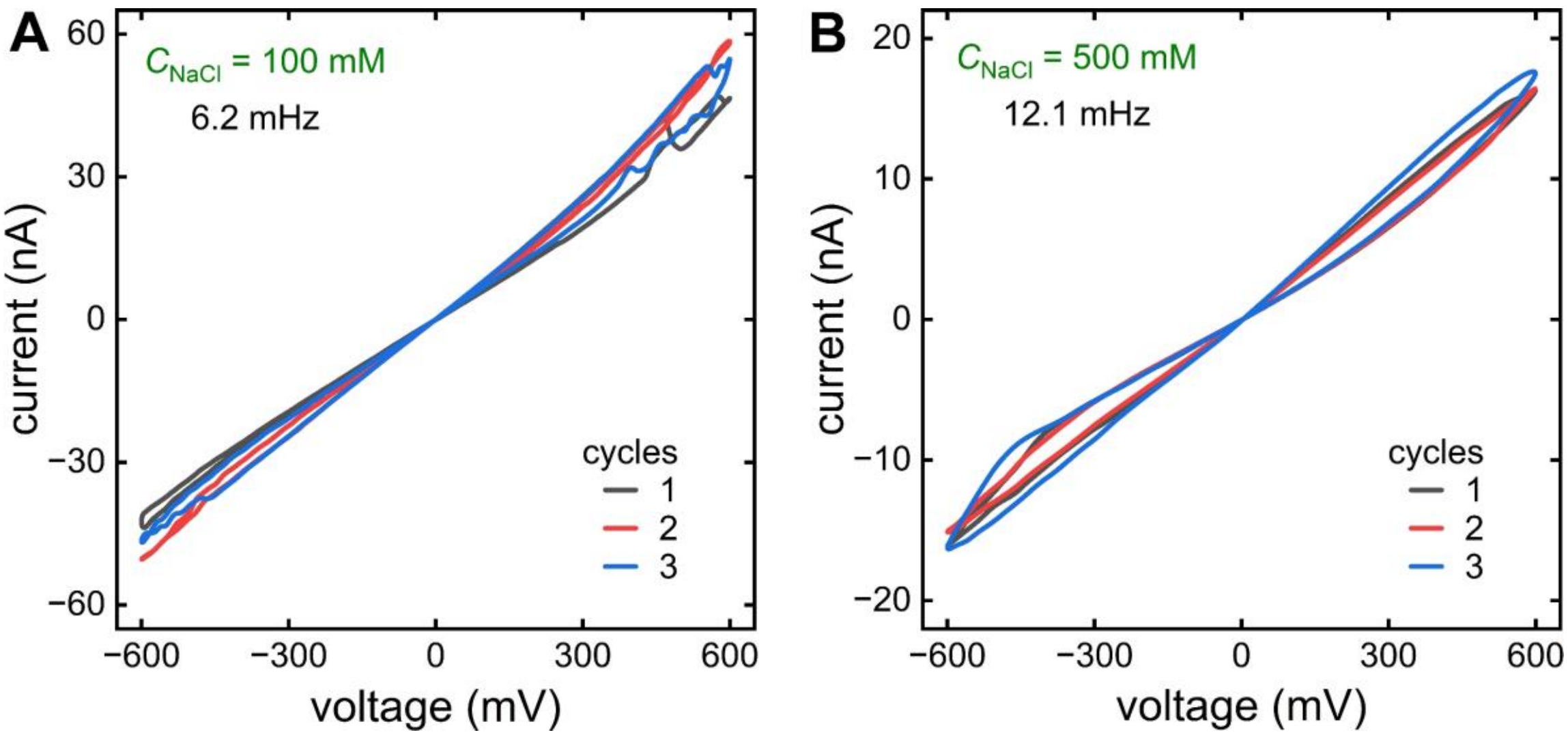

Fig. S23: Successive *I-V* loops. Successive *I-V* loops for (**A**) T_{CuMOF} = 15 nm with 100 mM NaCl at 6.2 mHz and (**B**) T_{CuMOF} = 20 nm with 500 mM at 12.1 mHz.

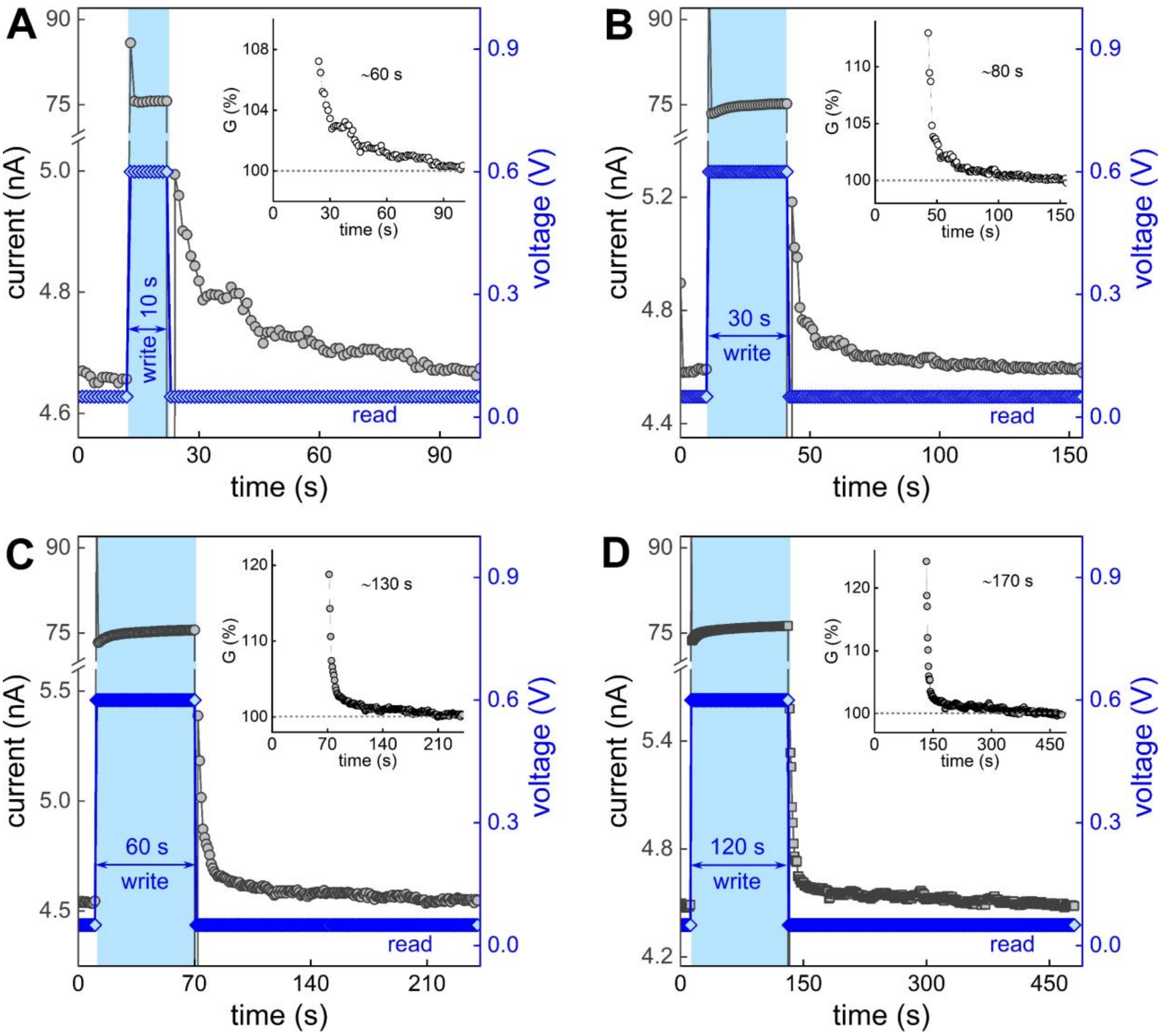

Fig S24. Plots showing the current vs time for a CuMOF device under writing durations of (**A**) 10 s, (**B**) 30 s, (**C**) 60 s and (**D**) 120 s. Insets in each panel show normalized conductance (*G* %) *vs* time after the write step, showing how long the device retains its written state. 500 mM NaCl is used as electrolyte.

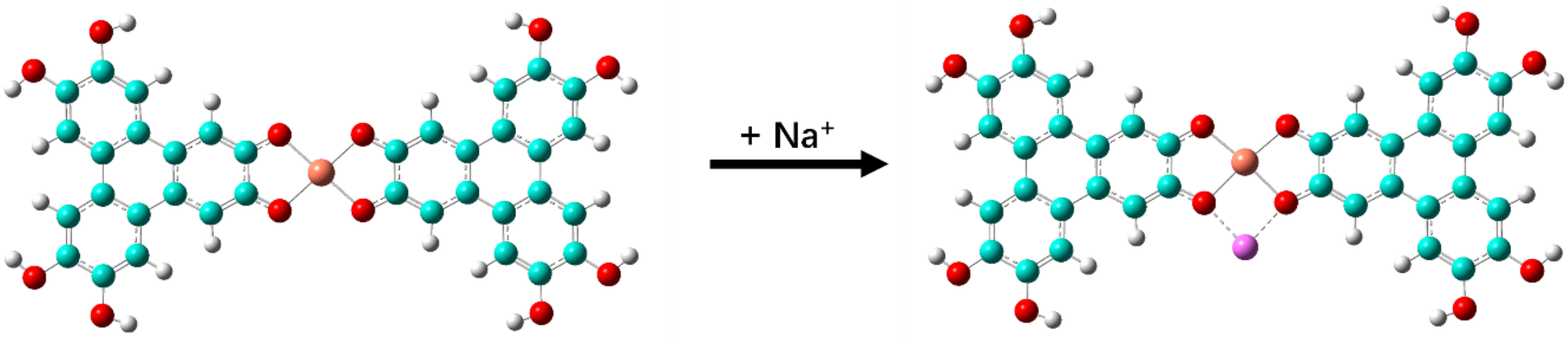

Fig. S25: Geometry optimization of CuMOF fragment for construction of the simulated ring-unit structure.

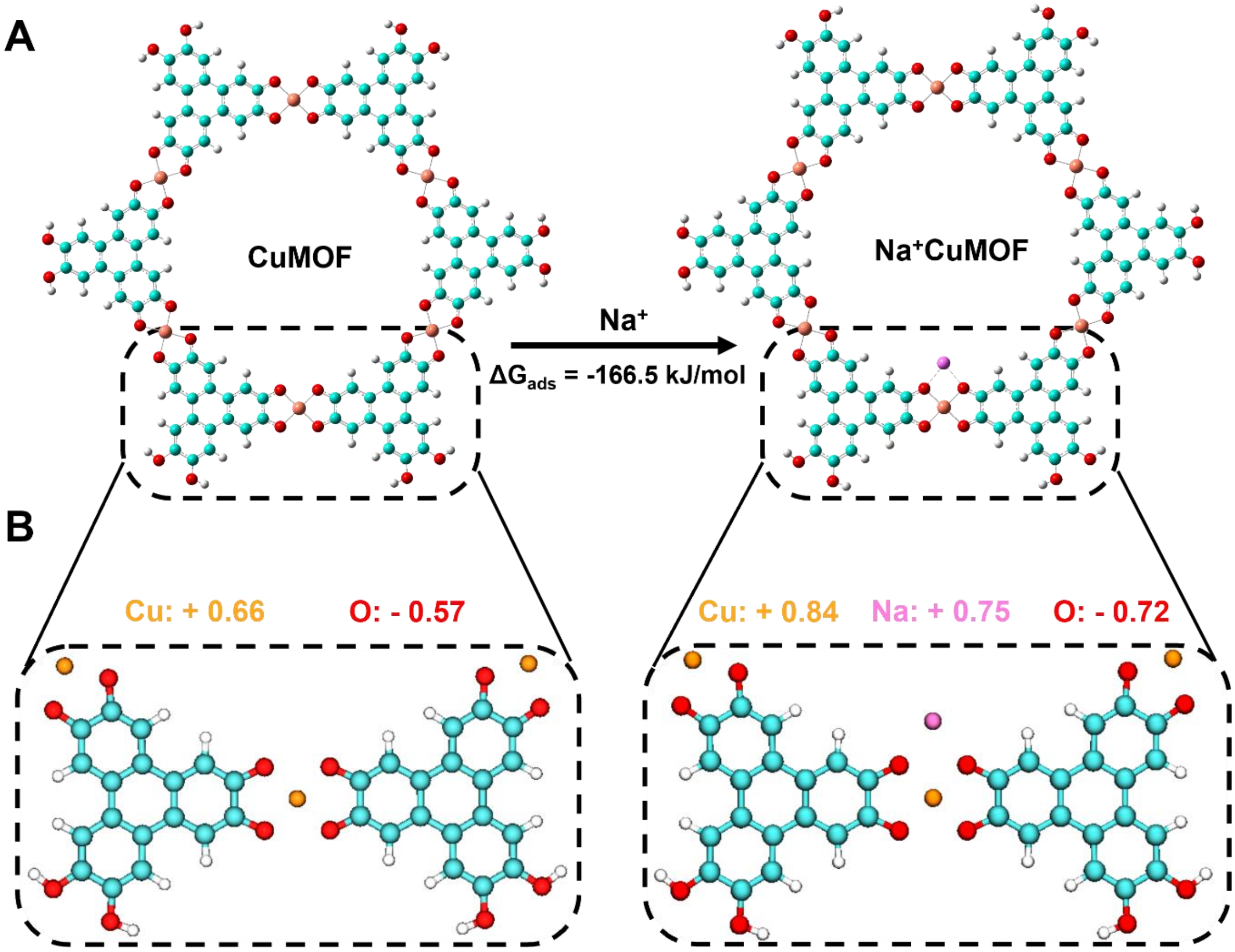

Fig. S26: DFT studies of sodium adsorption behavior. (A) Simulated structures of CuMOF before and after adsorption of Na^+, indicated with Gibbs Free Energy change of -166 $kJ.mol^{-1}$. **(B)** Charge distribution on copper, oxygen and sodium before and after adsorption of Na^+.

Table S3. Comparison of Nanofluidic transport features of different 2D materials based fluidic devices.

Material	Nanofluidic transport features and performances			Ref
	Ion selectivity	Osmotic energy	Ionic memory	
Graphene nanopores	K^+/Cl^- selectivity ratio over 100	-	-	[11]
Atomically thin graphene	Na^+/Cl^- selectivity 394	126 W/m^2 (1000-fold KCl gradient at 47 °C)	-	[12]
Single-layer MoS_2 nanopores	Max. transference number 0.92 for K^+ over Cl^-.	10^6 W/m^2 (KCl salt gradient)	-	[13]
Vermiculite nanochannel from ultrathin slicing	-	234.6 W/m^2 (10-fold concentration gradient)	-	[14]
2D slit nanochannels (MoS_2/Graphene)	-	-	Long-term memory spans from minutes to hours	[15]
MoS_2 nanopore	-	-	Short-term memory ranging from seconds to minutes and decay of 26.5 s- ~69.2 s	[16]
Slit 2D channels (hBN/MoS_2/ Graphene)	-	-	Multiple memory types. Retention up to ~74 hours in a bipolar type	[17]
MoS_2 SnS_2 nanosheets (stacked heterojunction)	-	-	Short-term: tens of seconds<br>Long-term: > 10^3 seconds (typically ~20–30 min depending on test)	[18]
rGO membrane embedded in epoxy resin	-	-	*Long memory retention.* (~50% synaptic enhancement and ~40% suppression persisting beyond 5 h without noticeable decay)	[19]
Vermiculite membrane	-	-	Switching of memory type	[20]
MoS_2 metallic composite membrane	-	6.7 W/m^2 (sea/river water gradient)	-	[21]
Nanoconfined hydrogel based membrane	High cation selectivity of 0.933 (500-fold NaCl gradient)	52.1 W/m^2 (500-fold NaCl gradient)	-	[22]
MoS_2- hydrophilic bacterial nanocellulose membrane	-	233 W/m^2 (50-fold concentration gradient at 343 K)	-	[23]
2D MOF/MXene membrane	-	>49 W/m^2 salt-lake/river water interface.	-	[24]

MOFs@MXene heterostructured membrane	Max. transference number > 0.9 for 10 gradient and > 0.7 for different gradient.	25.3 W/m^2 (10^6 gradient KCl)	-	[25]
Janus NP-MXene membrane	selectivity ratios Na^+/Mg^{2+} = 110.34 Cl^-/SO_4^{2-} = 91.45	85.1 W/m^2 (50-fold salinity gradient)	-	[26]
hBN/GO hybrid membrane	Max. transference number 1 for K^+ over Cl^-.	9.11 W/m^2 (50 gradient KCl with photo-enhanced)	-	[27]
2D polyimine membranes	Selectivity coefficient: 0.98 (Na^+/Cl^-) and 0.93 (K^+/Cl^-).	53 W/m^2 (NaCl gradient of river/seawater)		[28]
Oxygen-rich functionalized MXene	-	21.7 W/m^2 (natural seawater and river water)	-	[29]
UiO-66-NH_2 MOF	-	26.8 W/m^2 (100-fold KBr gradient)	-	[30]
TpEB@TpPa-SO3Na COF	-	210.1 W/m^2 (50-fold NaI gradient)	-	[31]
Kelp cellulose nanofibers/MXene membrane	High cation transference numbers (~0.93 at 50-fold gradient)	10.56 W/m^2 (natural seawater and simulated river water).		[32]
Cation-MXene / Cation holocellulose nanofiber composite film	-	1.09 W/m^2 under a 50-fold salinity gradient	-	[33]
Porphyrin- metal-organic framework-anodic aluminum oxide membrane	Cation selectivity of 0.92 (K^+/Cl^-)	7.74 W m^{-2} (3000-fold concentration gradient).	-	[34]
ZIF-8 on top of a UiO-66-NH_2 membrane	-	40.01 W/m^2 (500-fold concentration gradient)	-	[35]
2D CuMOF	Maximum transference number ~0.92 for Na^+ over Cl^- with T_{CuMOF} = 20 nm	12.34 W/m^2 (10 gradient NaCl with T_{CuMOF} = 9.5 nm)	Memory retention ~170 s	This work

11. References

[1] R. C. Borner, N. Boden, R. J. Bushby, R. Borner, A. N. Cammidge, R. Bushby, A. Cammidge, M. Jesudason, *Liq. Cryst.* **2006**, *33*, 1439-1448.

[2] O. Bikondoa, L. Bouchenoire, S. D. Brown, P. B. Thompson, D. Wermeille, C. A. Lucas, M. J. Cooper, T. P. Hase, *Phil. Trans. R. Soc. A* **2019**, *377*, 20180237.

[3] W. Humphrey, A. Dalke, K. Schulten, *J. Mol. Graph.* **1996**, *14*, 33-38.

[4] G. Ashiotis, A. Deschildre, Z. Nawaz, J. P. Wright, D. Karkoulis, F. E. Picca, J. Kieffer, *Appl. Crystallogr.* **2015**, *48*, 510-519.

[5] T. Lu, F. Chen, *J. Comput. Chem.* **2012**, *33*, 580-592.

[6] T. Lu, *J. Chem. Phy.* **2024**, *161*, 082503.

[7] L. H. Grey, H.-Y. Nie, M. C. Biesinger, *Appl. Surf. Sci.* **2024**, *653*, 159319.

[8] N. Fairley, V. Fernandez, M. Richard-Plouet, C. Guillot-Deudon, J. Walton, E. Smith, D. Flahaut, M. Greiner, M. Biesinger, S. Tougaard, *Appl. Surf. Sci. Adv.* **2021**, *5*, 100112.

[9] E. Secchi, A. Niguès, L. Jubin, A. Siria, L. Bocquet, *Phys. Rev. Lett.* **2016**, *116*, 154501.

[10] D. R. Lide, *CRC Handbook of Chemistry and Physics, Vol. 85*, CRC press, **2004**.

[11] R. C. Rollings, A. T. Kuan, J. A. Golovchenko, *Nat. Commun.* **2016**, *7*, 11408.

[12] Y. Fu, X. Guo, Y. Wang, X. Wang, J. Xue, *Nano Energy* **2019**, *57*, 783-790.

[13] J. Feng, M. Graf, K. Liu, D. Ovchinnikov, D. Dumcenco, M. Heiranian, V. Nandigana, N. R. Aluru, A. Kis, A. Radenovic, *Nature* **2016**, *536*, 197-200.

[14] A. Bhardwaj, R. K. Gogoi, W. J. Howard, E. Tillotson, S. Goutham, Y. You, T. Hashimoto, E. Janzen, J. H. Edgar, S. J. Haigh, *Adv. Funct. Mater.* **2024**, *34*, 2401988.

[15] P. Robin, T. Emmerich, A. Ismail, A. Niguès, Y. You, G.-H. Nam, A. Keerthi, A. Siria, A. Geim, B. Radha, *Science* **2023**, *379*, 161-167.

[16] R. Song, P. Wang, H. Zeng, S. Zhang, N. Wu, Y. Liu, P. Zhang, G. Xue, J. Tong, B. Li, *Nano Letters* **2025**, *25*, 5646-5655.

[17] A. Ismail, G.-H. Nam, A. Lokhandwala, S. V. Pandey, K. V. Saurav, Y. You, H. Jyothilal, S. Goutham, R. Sajja, A. Keerthi, *Nat. Commun.* **2025**, *16*, 7008.

[18] H. Lv, Y. Zhang, *Biosens. Bioelectron.* **2025**, *282*, 117496.

[19] X. Peng, G. Zhang, H. Tian, H. Jiao, C. Ye, H. Zhao, L. Zhai, D. Wang, Y. Zhang, Y. Xue, *Nano Letters* **2025**, *25*, 12905-12913.

[20] D. Biswabhusan, P. Animesh, L.-H. Yeh, K. Gopinadhan, *ACS Appl. Mater. Interfaces* **2024**, *16*, 69556-69563.

[21] C. Zhu, P. Liu, B. Niu, Y. Liu, W. Xin, W. Chen, X.-Y. Kong, Z. Zhang, L. Jiang, L. Wen, *J. Am. Chem. Soc.* **2021**, *143*, 1932-1940.

[22] Y.-C. Lin, H.-H. Chen, C.-W. Chu, L.-H. Yeh, *Nano Letters* **2024**, *24*, 11756-11762.

[23] X. Wang, Z. Wang, Z. Xue, Y. Fan, J. Yang, Q. Zhang, N. Yang, X. Meng, Y. Jin, S. Liu, *Energy Env. Sci.* **2024**, *17*, 6225-6233.

[24] W.-H. Lin, T.-Y. Huang, C.-H. Bai, C.-H. Hung, C.-A. Lung, W.-H. Hung, K. Gopinadhan, L.-H. Yeh, *Nano Energy* **2024**, *128*, 109924.

[25] B. Yao, Z. Fang, Y. Hu, Z. Ye, X. Peng, *J. Memb. Sci.* **2024**, *709*, 123116.

[26] H. Qian, H. Fan, P. Peng, Y. Du, X. Li, Y. Liu, F. Yang, Y. Zhou, Z. L. Wang, D. Wei, *Sci. Adv.* **2025**, *11*, eadx1184.

[27] D. Sun, W. Guo, S. Li, Y. Li, X. Yu, Z. Liu, *Small* **2026**, *22*, e13109.

[28] Z. Zhang, P. Bhauriyal, H. Sahabudeen, Z. Wang, X. Liu, M. Hambsch, S. C. Mannsfeld, R. Dong, T. Heine, X. Feng, *Nat. Commun.* **2022**, *13*, 3935.

[29] Z. Ren, Q. Zhang, J. Yin, P. Jia, W. Lu, Q. Yao, M. Deng, Y. Gao, N. Liu, *Adv. Funct. Mater.* **2024**, *34*, 2404410.

[30] Y.-C. Liu, L.-H. Yeh, M.-J. Zheng, K. C.-W. Wu, *Sci. Adv.* **2021**, *7*, eabe9924.
[31] L. Cao, I.-C. Chen, X. Liu, Z. Li, Z. Zhou, Z. Lai, *ACS Nano* **2022**, *16*, 18910-18920.
[32] R. Zhai, L. Jiang, Z. Chen, X. Zhang, J. Zhou, X. Ma, C. Teng, Y. Zhou, L. Jiang, *Adv. Funct. Mater.* **2024**, *34*, 2313914.
[33] J. Rao, Z. Lv, X. Yan, J. Pan, G. Chen, B. Lü, F. Peng, *Adv. Funct. Mater.* **2024**, *34*, 2309869.
[34] Z. Q. Li, G. L. Zhu, R. J. Mo, M. Y. Wu, X. L. Ding, L. Q. Huang, Z. Q. Wu, X. H. Xia, *Angew. Chem. Int. Ed.* **2022**, *61*, e202202698.
[35] R. K. Tonnah, M. Chai, M. Abdollahzadeh, H. Xiao, M. Mohammad, E. Hosseini, M. Zakertabrizi, D. Jarrahbashi, A. Asadi, A. Razmjou, *ACS Nano* **2023**, *17*, 12445-12457.
